# Multiple imputation methods for handling missing values in a longitudinal categorical variable with restrictions on transitions over time: a simulation study

**DOI:** 10.1186/s12874-018-0653-0

**Published:** 2019-01-10

**Authors:** Anurika Priyanjali De Silva, Margarita Moreno-Betancur, Alysha Madhu De Livera, Katherine Jane Lee, Julie Anne Simpson

**Affiliations:** 1 Centre for Epidemiology and Biostatistics, Melbourne School of Population and Global Health, University of Melbourne, Melbourne, Victoria Australia; 2 Clinical Epidemiology and Biostatistics Unit, Murdoch Childrens Research Institute, Royal Children’s Hospital, Melbourne, Victoria Australia; 3 Department of Epidemiology and Preventive Medicine, Monash University, Melbourne, Victoria Australia; 4 Department of Paediatrics, University of Melbourne, Melbourne, Victoria Australia

**Keywords:** Fully conditional specification, Longitudinal categorical data, Missing data, Multiple imputation, Multivariate normal imputation, Restricted transitions

## Abstract

**Background:**

Longitudinal categorical variables are sometimes restricted in terms of how individuals transition between categories over time. For example, with a time-dependent measure of smoking categorised as never-smoker, ex-smoker, and current-smoker, current-smokers or ex-smokers cannot transition to a never-smoker at a subsequent wave. These longitudinal variables often contain missing values, however, there is little guidance on whether these restrictions need to be accommodated when using multiple imputation methods. Multiply imputing such missing values, ignoring the restrictions, could lead to implausible transitions.

**Methods:**

We designed a simulation study based on the Longitudinal Study of Australian Children, where the target analysis was the association between (incomplete) maternal smoking and childhood obesity. We set varying proportions of data on maternal smoking to missing completely at random or missing at random. We compared the performance of fully conditional specification with multinomial and ordinal logistic imputation, and predictive mean matching, two-fold fully conditional specification, indicator based imputation under multivariate normal imputation with projected distance-based rounding, and continuous imputation under multivariate normal imputation with calibration, where each of these multiple imputation methods were applied, accounting for the restrictions using a semi-deterministic imputation procedure.

**Results:**

Overall, we observed reduced bias when applying multiple imputation methods with restrictions, and fully conditional specification with predictive mean matching performed the best. Applying fully conditional specification and two-fold fully conditional specification for imputing nominal variables based on multinomial logistic regression had severe convergence issues. Both imputation methods under multivariate normal imputation produced biased estimates when restrictions were not accommodated, however, we observed substantial reductions in bias when restrictions were applied with continuous imputation under multivariate normal imputation with calibration.

**Conclusion:**

In a similar longitudinal setting we recommend the use of fully conditional specification with predictive mean matching, with restrictions applied during the imputation stage.

**Electronic supplementary material:**

The online version of this article (10.1186/s12874-018-0653-0) contains supplementary material, which is available to authorized users.

## Background

The problem of missing data is prominent in longitudinal studies as these studies involve gathering information from respondents at multiple waves over a long period of time [1]. One approach for handling such missing data is multiple imputation (MI), which has become a frequently used method for handling missing data in observational epidemiological studies [2]. MI is a two stage process [3]. In the first stage, the incomplete dataset is replicated multiple times, with the missing values replaced by values drawn from an appropriate imputation model. In the second stage, the analysis of interest is performed on each of the imputed datasets and resulting parameter estimates are combined using Rubin’s rules [3]. Multivariate normal imputation (MVNI), and fully conditional specification (FCS), are widely available MI methods that have been used in longitudinal studies [4, 5] to impute missing values.

MVNI imputes missing values by fitting a joint imputation model for all the variables with missing data, assuming that these variables follow a multivariate normal distribution [6]. FCS uses univariate regression models fitted to each variable with missing data depending on the type of variable with missing data [7, 8]. When handling missing values in longitudinal data, standard implementations of MVNI and FCS can be applied by treating repeated measurements of the same variable at different time points as distinct variables, sometimes referred to as the “Just Another Variable” approach [9]. For example, measurements of quality of life at different time points are treated as separate variables. This needs to be done for all the longitudinal variables. This approach does not explicitly model the longitudinal structure of the data, although it does allow for the correlations between the repeated measurements. The two-fold FCS algorithm is a recently proposed version of FCS that takes into consideration the longitudinal structure of the data by imputing missing values in a variable at a certain time point, using information only from the specific time point and immediately adjacent time points [9, 10]. Two-fold FCS may help to reduce convergence issues encountered with FCS in longitudinal studies with large numbers of waves and incomplete variables [9].

In many epidemiological studies, variables are collected that involve several restrictions. One example is that of restricted-transition variables. These are categorical variables where the set of possible future states depends on its current and previous states. For example, with a time-dependent measure of smoking categorised as never-, ex-, and current-smoker, current- or ex-smokers cannot transition to a never-smoker at a subsequent wave. Oral contraceptive use measured repeatedly as a never-user, ex-user or current-user is another example of a time-dependent variable which is restricted such that an ex- or current-user cannot transition into a never-user at a subsequent wave. However, never-users may start using oral contraceptives at any time.

Guidance on how MI methods should be applied for handling missing data in such variables is limited in the statistical literature. For incomplete smoking data (non-, ex- and current-smoker), Welch et al. [9] focused on a simulation scenario where non-smokers at baseline did not transition into other smoking categories, and used deterministic imputation for the non-smoking category in this simulation study. Specifically, all respondents observed as non-smokers at any of the time points, were imputed as non-smokers for missing time points. Missing values for the remaining respondents were imputed stochastically, as either a current-smoker or ex-smoker [9]. Although this semi-deterministic approach is appealing, it may not always be appropriate as in real-world situations some non-smokers may start smoking. Similarly, in the contraceptive use example, never-users may start using oral contraceptives over time. Another simulation study by Kalaycioglu et al. [5] explored a number of scenarios for handling missing values in longitudinal data, including a categorical treatment variable, which had transition restrictions. However, little information was available on how missing values were handled in this variable.

While the primary goal of MI is to obtain valid inferences, and not to replace the actual missing values per se [11], it is important to assess the impact of implausible imputation values on the parameter estimates of interest [6, 7, 12]. Therefore, the aim of this paper was to evaluate the performance of possible MI approaches (namely MVNI, FCS, and two-fold FCS algorithm) for handling missing values in a longitudinal categorical variable with restrictions on transitions over time. We report the findings of a case study from the Longitudinal Study of Australian Children (LSAC), and a simulation study based on the LSAC [13] where approximately 65% of data on maternal smoking were set to missing completely at random (MCAR) or missing at random (MAR). In this study, maternal smoking was a time-dependent categorical exposure variable with restrictions, measured repeatedly over six time points.

## Methods

### Motivating example: Longitudinal study of Australian children (LSAC)

The Longitudinal Study of Australian Children (LSAC) is a prospective study of 10,000 children, involving two cohorts, the infant cohort (B) and the child cohort (K). Data collected at six time points, from 2004 to 2014 [13] was available for this study. LSAC obtained written informed consent from the caregiver on behalf of each of the study children, as the children were minors at the time of data collection and was approved by the Australian Institute of Family Studies Ethics Committee.

### Epidemiological analysis of interest

Childhood obesity is a growing epidemic in most developed countries, and a common problem among Australian children [14]. Many severe health diseases are attributable to childhood obesity [15]. Importantly, exposure to maternal smoking has been found to be an important risk factor of childhood obesity [16–19]. The motivating example for our simulation study was to quantify the relationship between exposure to maternal smoking and body mass index (BMI).

#### Target analysis model

**Fig. 1:**
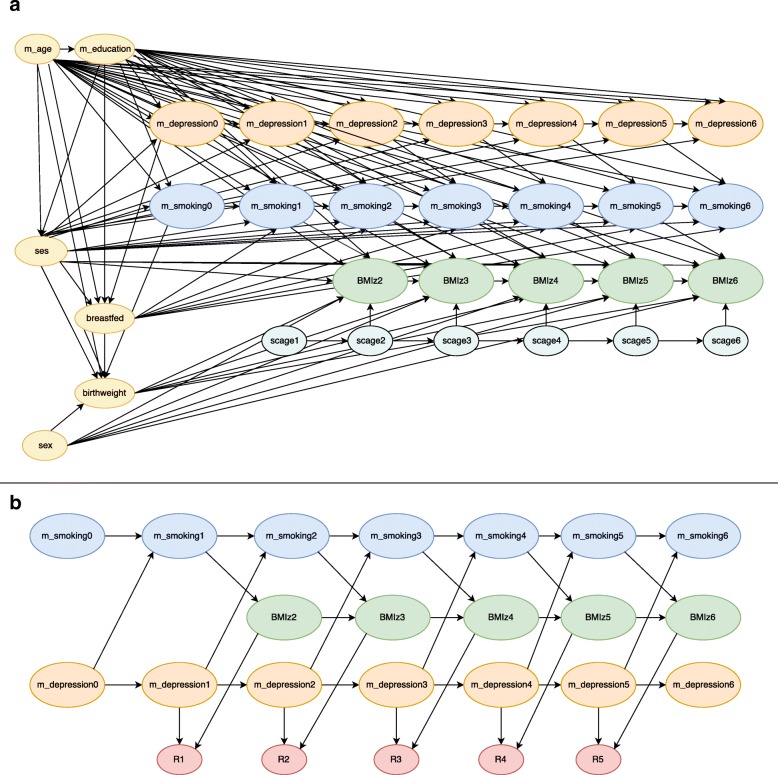
**a**) Causal diagram for the association between maternal smoking and subsequent body mass index (BMI) for age z-scores developed based on the literature; m_age, maternal age at child birth; m_education, maternal education; sex, study child’s sex; birthweight, study child’s birth weight; ses, family socio-economic status z-score; breastfed, breastfeeding patterns; BMIz2-BMIz6, study child’s BMI for age z-scores at waves 2 to 6; m_smoking0-m_smoking6, maternal smoking at waves 0 to 6; m_depression0-m_depression6, maternal depression at waves 0 to 6; **b**) Causal diagram for MAR missingness. R_j_ is an indicator variable of missingness where maternal smoking at wave j were assigned to missing if R_j_ = 1. Only variables required to model the MAR missingness are shown in the figure

The analysis of interest was the association between maternal smoking measured at one wave and BMI for age z-scores (BMIz) measured at the subsequent wave, estimated using a linear mixed-effects model with a random intercept and adjusted for child’s current age, birthweight, and sex, breastfeeding, maternal age at child birth, maternal education, and family socio-economic status (see Eq. 1 and Table 1 for description of the variables, and Fig. 1a for the causal diagram).

1 $$ {BMIz}_{i,j}=\left({\beta}_0+{b}_{0i}\right)+{\sum}_{a=1}^2{\beta}_{1,a}\left[m\_ smoki\mathrm{n}{g}_{i,j-1}=a\right]+{\beta}_2{scage}_{ij}+{\beta}_3\left[{breastfed}_i=1\right]+{\beta}_4m\_{age}_i+{\beta}_5\left[m\_{education}_i=1\right]+{\beta}_6{birthweight}_i+{\beta}_7\left[{sex}_i=1\right]+{\beta}_8{ses}_i+{\in}_{ij} $$

where *i* = 1,..., *N*, and *N* = 1000 for waves j = 1,…,6; ∈_*ij*_ is identically and independently distributed as ${\in}_{ij}\sim N\ \left(0,{\sigma}_{\in}^2\right)$; a = 0 (never-smoker – reference category), 1 (ex-smoker) and 2 (current-smoker); *β*_0_ is the population parameter for mean BMIz when other covariates are set to zero and *b*_0*i*_ is the random intercept for individual i, assumed to be normally distributed with mean zero, and constant variance; *β*_1_ − *β*_*8*_ are the population parameters for the mean change in BMIz associated with the covariates.

**Table 1:** Description of variables from the Longitudinal Study of Australian Children used in the simulation study for respondent i at wave j

Variable	Type	Grouping/Units	Label
Study child’s BMI for age^a^	Continuous	z-score	BMIz_ij_
Maternal smoking	Categorical	0 = Never-smoker 1 = Ex-smoker	m_smoking_ij_
		2 = Current-smoker	
Maternal depression	Categorical	0 = No	m_depression_ij_
		1 = Yes	
Maternal age at child birth	Continuous	Years	m_age_i_
Maternal education	Categorical	0 = Not completed 1 = Completed	m_education_i_
Breastfeeding	Categorical	0 = No 1 = Yes	breastfed_i_
Family socio-economic status	Continuous	z-score	ses_i_
Study child’s sex	Categorical	0 = Female 1 = Male	sex_i_
Study child’s birth weight	Continuous	kilograms	birthweight_i_
Study child’s age	Continuous	Months	scage_ij_

Abbreviations: BMI, body mass index

^a^Raw BMI measurements converted into BMI for age z-scores using the 2000 Centre for Disease Control growth charts

### Simulation of complete data

The simulation study was based on six waves of the LSAC infant cohort, which had a participation of 5107 children at wave 1 (see Additional file 1: Table S1). Data were generated as specified below based on the casual diagram in Fig. 1a. This process was repeated to generate 1000 complete datasets. A detailed description of the simulation procedure is provided in the Additional file 1.

After simulating the time-independent variables, the time-dependent exposure (m_smoking) and outcome (BMIz) were simulated as follows:

- Maternal smoking at wave 0 (i.e. during pregnancy) (m_smoking_i,0_) was generated from a multinomial logistic regression model:

2 $$ logit\ \left\{\Pr \left(m\_{smoking}_{i,0}=a\right)\right\}={\upeta}_{0,\mathrm{a}}+{\upeta}_{1,\mathrm{a}}m\_{age}_i+{\upeta}_{2,\mathrm{a}}\left[m\_{education}_i=1\right]+{\upeta}_{3,\mathrm{a}}{ses}_i $$

- Maternal smoking at waves j = 1,…,6 (m_smoking_i,j_) was generated in two stages.
- Stage 1: Maternal smoking was generated for respondents who were never-smokers at the previous wave using the multinomial logistic regression model:

3 $$ logit\ \left\{\Pr \left(m\_{smoking}_{i,j}=b|\ m\_{smoking}_{i,j-1}=0\right)\right\}={\upzeta}_{0,\mathrm{a}}+{\upzeta}_{1,\mathrm{a}}m\_{age}_i+{\upzeta}_{2,\mathrm{a}}\left[m\_{education}_i=1\right]+{\upzeta}_{3,\mathrm{a}}\left[m\_{depression}_{i,j-1}=1\right]+{\upzeta}_{4,\mathrm{a}}{ses}_i $$

- Stage 2: Maternal smoking for the remaining respondents (current- or ex-smoker) was generated using the logistic regression model:

4 $$ logit\ \left\{\Pr \left(m\_{smoking}_{i,j}=2|\ m\_{smoking}_{i,j-1}\ne 0\right)\right\}={\upkappa}_0+{\upkappa}_1m\_{age}_i+{\upkappa}_2\left[m\_{education}_i=1\right]+{\upkappa}_3\left[m\_{depression}_{i,j-1}=1\right]+{\upkappa}_4\left[m\_{smoking}_{i,j-1}=2\right]+{\upkappa}_5{ses}_i $$

- BMI for age z-scores (BMIz_i,j_) were generated for waves j = 2,…,6 using the linear mixed-effects model in Eq. 1 so that the chosen values for β_1,a_ (a = 1, 2) of Eq. 1 are the true values for the parameters of interest.

We considered β_1,1_ = 0.10 and β_1,2_ = 0.15. In general, parameter values used in the simulation process were chosen to mimic the LSAC data (see Additional file 1: Table S2).

### Generation of missing data

For each of the 1000 simulated datasets, and at each wave, maternal smoking values were randomly assigned to missing such that for some individuals, measurements in all subsequent waves were also missing (i.e. dropout) while for others future values of maternal smoking could be missing or observed (i.e. intermittent missingness). The proportions of missingness per waves were as in the LSAC (see Additional file: Fig. S1). Missingness was generated under an MCAR mechanism, or either of two MAR mechanisms, representing weak or strong associations between the probability of missingness and predictors of missingness (see Table 2).

**Table 2:** Specifications of the parameters in the logistic regression models used to impose missing data under the missing at random scenarios

Variable	Odds Ratio			
	MAR (weak)		MAR (strong)^a^	
	Model A Equation 5^b^	Model B Equation 6^b^	Model A Equation 5^b^	Model B Equation 6^b^
Maternal depression at wave j	exp(*ν*_1_) = 1.67	exp(*ω*_1_) = 1.61	exp(*ν*_1_) = 2.80	exp(*ω*_1_) = 2.70
BMI for age z-scores at wave j + 1	exp(*ν*_2_) = 1.64	exp(*ω*_2_) = 1.58	exp(*ν*_2_) = 2.60	exp(*ω*_2_) = 2.50

Abbreviations: BMI, body mass index; exp., exponential; MAR, missing at random

^a^Odds ratio for MAR (Strong) = square of the Odds ratio for MAR (Weak)

^b^Models A and B represent the logistic regression models used to generate missingness in maternal smoking from waves 1–5 under MAR, in all subsequent waves and intermittently respectively

Specifically, under each MAR mechanism, it was assumed the probability of missingness in maternal smoking followed a logistic regression model dependent on BMIz and the auxiliary variable maternal depression (Fig. 1b). The d-separation criterion [20] was used to show that missingness is independent of unobserved data conditional on maternal depression at wave j (m_depression_j_) and BMIz measured at the subsequent wave (BMIz_j + 1_) that is, the MAR assumption holds given these variables (see Additional file 1). The models used to generate missing values in maternal smoking were:

Model A: missing for all subsequent waves

$$ logit\left\{\Pr \left({R}_{i,1}=1\right)\right\}={\nu}_{0,1}+{\nu}_1\left[m\_{depression}_{i,1}=1\right]+{\nu}_2{BMIz}_{i,2} $$

$$ \Pr \left({R}_{i,j}=1|{R}_{i,j-1}=1\right)=1;2\le j\le 5 $$

5 $$ logit\left\{\Pr \left({R}_{i,j}=1|{R}_{i,j-1}=0\right)\right\}={\nu}_{0,j}+{\nu}_1\left[m\_{depression}_{i,j}=1\right]+{\nu}_2{BMIz}_{i,j+1};2\le j\le 5 $$

Model A introduces monotone missingness, such that, if the measurement at wave *j* is specified as missing using model A, then the individual will have measurements missing for all subsequent waves *j* + 1, … , 5.

Model B: intermittent missingness between waves j-1 and j

$$ logit\left\{\Pr \left({R}_{i,j}=1\right)\right\}={\omega}_{0,j}+{\omega}_1\left[m\_{depression}_{i,j}=1\right]+{\omega}_2{BMIz}_{i,j+1};j\le 5 $$

(6)

where *R*_*i*, *j*_ is an indicator variable of missingness, and maternal smoking was assigned to missing for respondent i at wave j if *R*_*i*, *j*_ = 1.

Model B was only applied to the respondents who were not specified as missing using model A. The strong MAR scenario was obtained by doubling the log of the odds ratios used in the weak MAR scenario (see Table 2 for parameter values).

For each mechanism (MCAR or MAR), the overall missingness proportion for maternal smoking was set at 45 % and 65%, representing realistic and extreme scenarios respectively [21], resulting in 6 simulation scenarios.

### Methods to handle missing data

For comparison with MI methods, we first performed a complete case analysis (CCA), excluding all respondents with missing values for maternal smoking at any of the 5 waves, and an available case analysis (ACA), including available data at each wave in the analyses [22]. These approaches are commonly used due to simplicity [2, 22–24]. CCA and ACA are expected to produce biased estimates under the MAR scenarios explored in this study. Both CCA and ACA condition on the missingness indicator *R*_*j*_ (see Fig. 1b). This missingness indicator is a collider as it lies in the pathway ‘m_depression_j_→*R*_*j*_← BMIz_j + 1_’, opening a backdoor path between the exposure and outcome of interest that is not blocked in the analysis model given that maternal depression is an auxiliary variable not included in the target analysis. Therefore, in principle we expect biased estimates under CCA and ACA [25], although this bias may be small.

We then assessed three MI methods, MVNI, FCS, and two-fold FCS, to multiply impute missing values in maternal smoking at waves 1 to 5. Given that the missingness mechanism generated satisfies the MAR assumption given m_depression_j_ and BMIz_j + 1_, as explained previously, we expect in principle that appropriate MI methods incorporating the target analysis variables as well as the auxiliary maternal depression variable to produce unbiased estimates under the missing data scenarios considered. Specifically, we considered two versions of each of these MI methods; the standard version, and the restriction-adapted version that accounts for restrictions in transitions over time.

#### Standard version

In the standard implementation of MVNI and FCS, repeated measurements of maternal smoking were included as distinct variables in the imputation model (i.e. one variable for each time point). This ‘single-level’ imputation was used to impute missing data at all the time points. The correlation between the repeated measures is captured in this approach [4, 5], However, treating repeated measurements of the same variable as distinct variables fails to account for the temporal ordering of the data which may affect imputation [9].

With MVNI, due to the assumption of multivariate normality, the imputed values for maternal smoking could take non-integer values. Therefore, we used two methods for imputation; maternal smoking imputed as indicators using MVNI, followed by projected distance-based rounding (indicator-PDBR) [26], and maternal smoking imputed as a continuous variable using MVNI, followed by calibration (continuous-calibration) [27, 28], to re-categorise imputed values into the original categories (see Additional file 1, Figure S2 and S3).

Within the FCS framework we considered three univariate imputation methods: multinomial logistic regression, ordinal logistic regression (treating the smoking variable as continuous based on the numerical codes 0, 1, 2), and predictive mean matching (PMM) (using a linear prediction model to obtain predicted values and k = 5 and 10 for randomly drawing from k^th^ nearest observed values to the predicted value) [29].

With the two-fold FCS algorithm, missing values in maternal smoking were imputed using information from only specific and immediately adjacent time points, and assuming a multinomial logistic imputation model (ordinal logistic regression is not available in current implementation of two-fold FCS) [30].

We used a linear mixed-effects model with a random intercept as our analysis model. Even though we used a multilevel analysis model, missing data were imputed using single-level fixed-effect imputation methods. These single-level fixed-effect MI methods allow an unstructured correlation structure between the repeated measurements. This indicates that no unnecessary assumptions are made about the correlations, which makes the single-level fixed-effect MI methods more general than a multilevel MI method. Furthermore, all imputation models included all variables in the analysis model as predictors, as well as the time-dependent auxiliary variable maternal depression [31]. Hence the MI methods considered are approximately compatible with the analysis model. Even though single-level fixed-effect MI may lead to increased precision, the statistical literature has highlighted limitations of this method: it can inflate the sampling variance, lead to low coverage probabilities, and may be computationally demanding. These issues are discussed by Enders et al. [32].

#### Restriction-adapted version

**Fig. 2:**
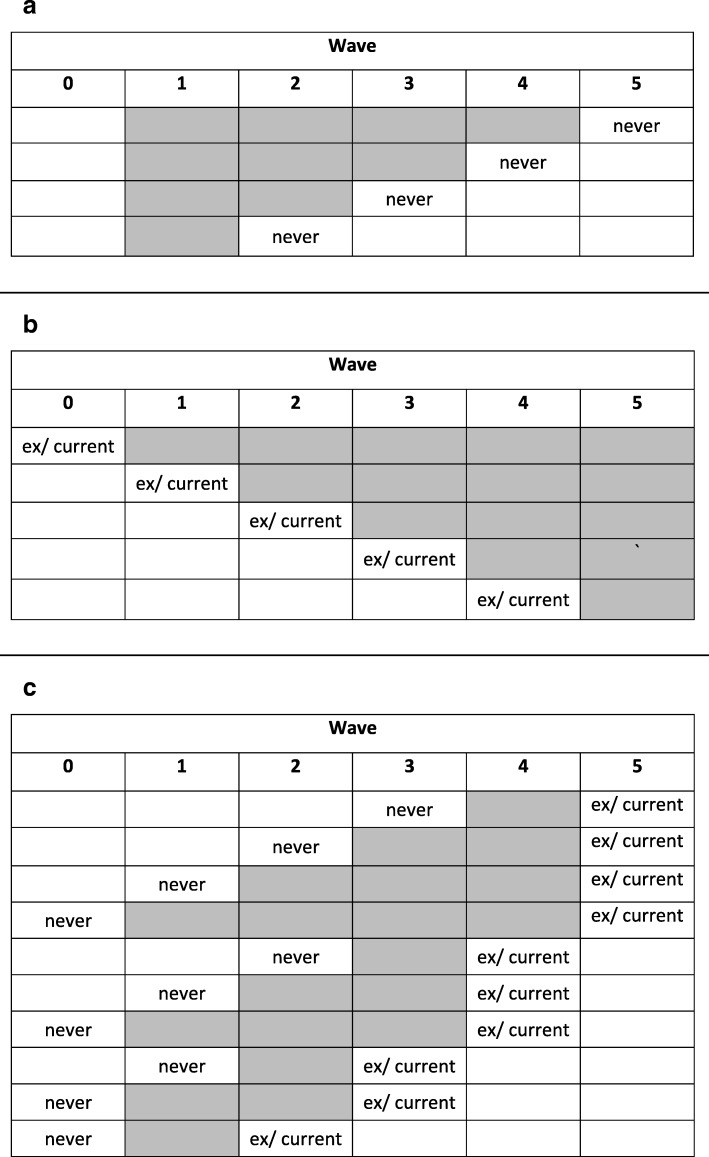
**a**) Scenarios to be imputed under stage 1 of the restriction process (never-smoker); **b**) Scenarios to be imputed under stage 2 of the restriction process (ex- or current-smoker); **c**) Scenarios to be imputed under stage 3 of the restriction process (never-, ex- or current-smoker); Grey boxes refer to data to be imputed at each stage if incomplete

We used a semi-deterministic approach, where missing values in maternal smoking at waves 1 to 5 were imputed according to a three-stage process, as follows:

- Stage 1: If a respondent was observed as a never-smoker at a specific wave, any missing values in all previous waves were deterministically assigned to be a never-smoker (Fig. 2a).
- Stage 2: If a respondent was observed as a current- or ex-smoker at a specific wave, any missing values in all subsequent waves were imputed stochastically as current- or ex-smokers (i.e. as a binary variable) (Fig. 2b).
- Stage 3: For the remaining scenarios (Fig. 2c), the missing values were imputed stochastically as never-, current- or ex-smokers.

In stage 3, it is inevitable that a small proportion of imputed values will violate the restrictions. However, we accepted these implausible values as it would be difficult to further introduce restrictions within the already existing restrictions.

### Performance measures for evaluating different methods

We estimated the target analysis parameters (β_1, a_ (a = 1,2) of Eq. 1) by fitting the linear mixed-effects model in Eq. 1.

We compared the performances of CCA, ACA, and the different MI methods (standard and restriction-adapted versions) using the absolute bias (difference between true value and average of MI estimates calculated from 1000 simulations); empirical standard error (square root of variance of 1000 estimates); and coverage of 95% confidence interval (proportion of simulated datasets in which the true parameter value was contained in the estimated 95% confidence interval). The relative bias (bias relative to true parameter value), the model-based standard error (average of standard errors of 1000 estimates) and mean square error (MSE), which is a combined measure of bias and efficiency [33], were also reported. The Monte Carlo errors for the MI estimates were used to assess the variation in estimated parameters across the simulations [34].

### Case study analysis

In addition to the simulation study, we also provide an empirical comparison of the methods considered, using the data from the LSAC infant cohort. We used wave-specific measures of whether the mother currently smoked or not to derive the never-smoker, ex-smoker and current-smoker at waves 1 through 6 (see Additional file 1).

Stata 13 statistical software [35] was used for all analyses.

## Results

### Results from simulation study

The standard and two-fold FCS methods with multinomial logistic regression imputation models failed to converge in all 1000 simulations for each of the 6 simulation scenarios. Standard FCS with ordinal logistic regression imputation showed extremely high non-convergence rates (up to 95%). The results for standard and two-fold FCS methods with multinomial logistic regression imputation, and FCS with ordinal regression are no longer considered in the following description of the results.

**Fig. 3:**
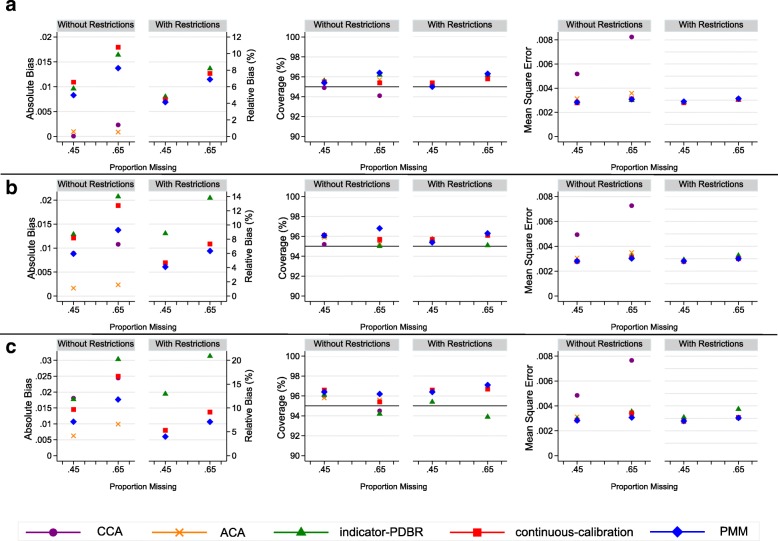
Absolute and Relative bias (%), Coverage (%), and Mean square error for complete case analysis (CCA), available case analysis (ACA), indicator based imputation using multivariate normal imputation with projected distance-based rounding (indicator-PDBR), imputation as a continuous variable using multivariate normal imputation with calibration (continuous-calibration), and predictive mean matching (PMM) for handling increasing proportions of missing data (0.45, 0.65), for the parameter estimates for current-smokers relative to never-smokers for the simulation study, when data are a) missing completely at random; b) missing at random (weak); c) missing at random (strong). Results are not shown for fully conditional specification with multinomial and ordinal logistic imputation and two-fold fully conditional specification methods because the imputation models failed to converge in some or all of the simulations. Minimal differences were observed between the results of predictive mean matching with 5 and 10 nearest observations. Therefore, only the results for this method with 5 nearest observations are presented. Complete case analysis and available case analysis are presented under without restrictions for comparison purposes only

**Fig. 4:**
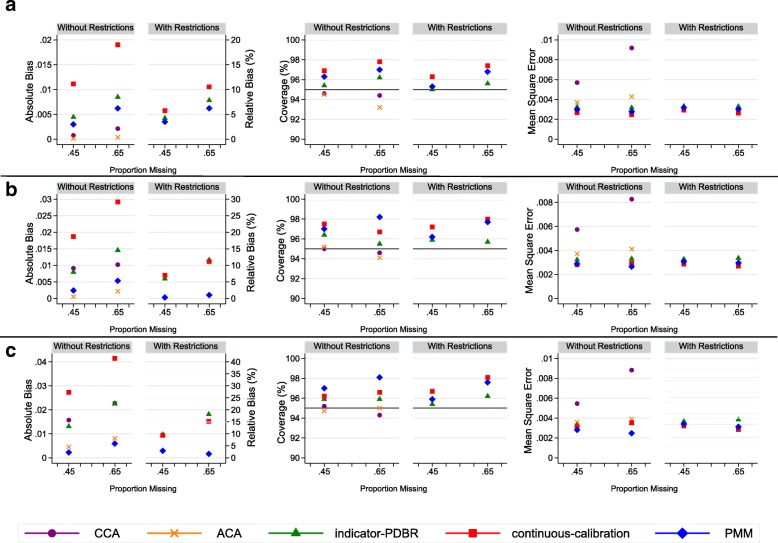
Absolute bias, Relative bias (%), Coverage (%), and Mean square error for complete case analysis (CCA), available case analysis (ACA), indicator based imputation using multivariate normal imputation with projected distance-based rounding (indicator-PDBR), imputation as a continuous variable using multivariate normal imputation with calibration (continuous-calibration), and predictive mean matching (PMM) for handling increasing proportions of missing data (0.45, 0.65), for the parameter estimates for ex-smokers relative to never-smokers for the simulation study, when data are a) missing completely at random; b) missing at random (weak); c) missing at random (strong). Results are not shown for fully conditional specification with multinomial and ordinal logistic imputation and two-fold fully conditional specification methods because the imputation models failed to converge in some or all of the simulations. Minimal differences were observed between the results of predictive mean matching with 5 and 10 nearest observations. Therefore, only the results for this method with 5 nearest observations are presented. Complete case analysis and available case analysis are presented under without restrictions for comparison purposes only

As expected we observed minimal bias under CCA and ACA when data were MCAR, with the relative bias not exceeding 3% (Figs. 3a and 4a). In both MCAR scenarios, the MI methods (FCS with PMM, indicator-PDBR and continuous-calibration) produced more biased estimates than CCA and ACA (a minimum relative bias of 0.05% produced by CCA and for the MI methods a maximum relative bias of 19.01% produced by continuous-calibration without restrictions). However, when data were MAR, the CCA resulted in more bias than most MI approaches, particularly in the strong MAR scenario (Figs. 3c and 4c). ACA still produced low bias (relative bias less than 10%) (Additional file 1: Tables S4-S7) and performed better than all of the MI methods in nearly all scenarios. FCS with PMM performed better than the other MI methods in terms of bias, in most MAR scenarios, under the standard implementation of MI, and we observed further reductions in bias under the restriction-adapted version, with the relative bias remaining under 10% for all missingness scenarios. Both imputation approaches under MVNI resulted in a high level of bias under the standard version. Convergence issues in up to 0.3% of the simulations across the 6 missingness mechanisms when no restrictions were applied were observed with indicator-PDBR. Little difference was observed in bias for indicator-PDBR with restrictions compared to the standard version; however, the non-convergence was lowered to a maximum of 0.1% across the 6 scenarios. We observed substantial reductions in bias for continuous-calibration (a reduction of relative bias of up to 26%, Fig. 4c) under the restriction-adapted version compared to the standard implementation.

For all MI methods with no issues of convergence, we found substantial gains in precision compared to CCA. However, for ACA we observed slightly larger empirical standard errors compared to these MI approaches. Across these MI methods, there was minimal difference in precision irrespective of the imputation approach and whether it was applied with or without restrictions. The gain in precision for MI compared to CCA and ACA was also reflected in the MSE, in which the MI methods produced a substantially lower MSE compared to CCA, and a slightly lower MSE compared to ACA. FCS with PMM performed better in terms of MSE than the other imputation approaches in most missingness scenarios when no restrictions were applied, however, we did not observe much difference in MSE when restrictions were applied.

The coverage was within 93.6 and 96.4% for the nominal level of 95% (expected range for coverage based on 1000 simulations) for most scenarios. However, a slight over-coverage was reported by both continuous-calibration and FCS with PMM for parameter estimates corresponding to ex-smokers relative to never-smokers, under both standard and restriction-adapted versions.

### Results from case study

Similar to the simulation study, the multinomial and ordinal logistic imputation models fitted under the FCS methods (both with and without restrictions) did not converge. Additionally, indicator-PDBR with restrictions, which showed some convergence issues in the simulation study, did not converge with the real data.

**Fig. 5:**
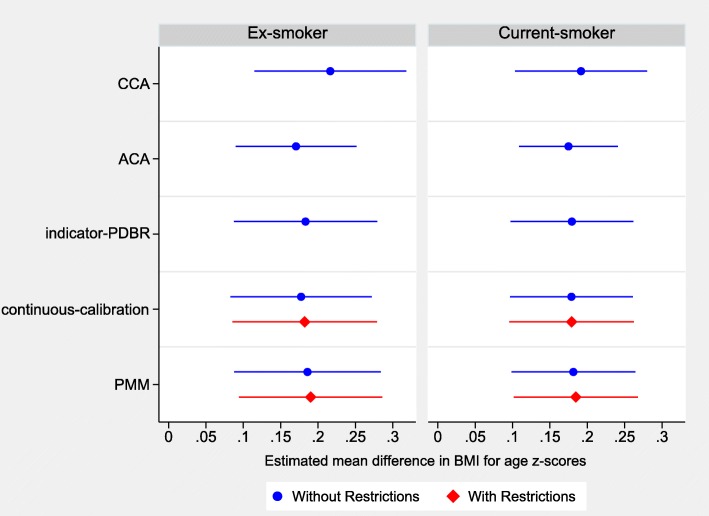
Estimated mean difference in body mass index (BMI) for age z-scores and 95% confidence intervals for ex-smokers and current-smokers compared to never-smokers for the case study analysis obtained from a random intercept linear mixed effects model using different methods^a^ for handling missing data in maternal smoking. ACA, available case analysis; CCA, complete case analysis; continuous-calibration, imputation as a continuous variable using multivariate normal imputation with calibration; indicator-PDBR, indicator based imputation using multivariate normal imputation with projected distance-based rounding; PMM^b^, predictive mean matching. ^a^ Results are not shown for indicator-PDBR with restrictions, fully conditional specification with multinomial and ordinal logistic imputation, and two-fold fully conditional specification methods because the imputation models failed to converge. ^b^Minimal differences were observed between the results of predictive mean matching with 5 and 10 nearest observations. Therefore, only the results for this method with 5 nearest observations are presented

As shown in Fig. 5, the CCA produced slightly large estimates for the mean differences and wider confidence intervals compared to the ACA and the MI methods that converged. The ACA gave smaller standard errors and narrower confidence intervals than all MI methods. Continuous-calibration and FCS with PMM were the only MI methods with restrictions that converged. We observed minimal differences in the estimates and confidence intervals when these methods were used with restrictions compared to without restrictions (Additional file 1: Table S3).

## Discussion

We compared the performance of MI methods, MVNI, FCS, and two-fold FCS, applied with and without restrictions, in addition to CCA and ACA, for handling missing data in a categorical variable with restrictions over time. We considered 6 different scenarios of missing data in maternal smoking, a longitudinal categorical exposure with three levels; never-smoker, ex-smoker and current-smoker, where an ex- or current-smoker at a specific wave is restricted from transitioning into a never-smoker.

Consistent with previously published studies [9, 21, 36–38], CCA and ACA produced negligible bias under MCAR. CCA excluded all individuals with missing data in at least one wave from the analysis. Missing data in maternal smoking were generated such that missingness was dependent on the outcome, BMI for age z-scores, after conditioning on the variables of the target analysis model. Therefore, as expected CCA produced biased estimates when data were MAR, with larger bias in the stronger MAR scenario. In contrast, in nearly all missingness scenarios investigated, ACA produced less biased estimates than MI without restrictions. This may be due to ACA accounting for most of the missingness mechanism due to the correlation between the repeated measurements. The imputation of implausible transitions under standard MI without restrictions is a possible reason for why this method produced more biased estimates than ACA. Furthermore, standard MVNI and FCS methods do not account for the temporal ordering of the repeated measurements as they treat repeated measurements of the same variable as distinct variables [9], which may explain the under-performance. However, simulation studies by Kalaycioglu et al. [5] and De Silva et al. [4] have shown that both MVNI and FCS may not be to susceptible to this issue as they have both been shown to have very good performance when including as much information as possible (i.e. all the repeated measurements) in the imputation model, as implemented in our study. Conversely, Kalaycioglu et al. [5] reported more biased estimates using ACA compared with MI without restrictions in the presence of multiple longitudinal variables with missing data, many of which were not restricted. In terms of precision, we observed substantial and slight gains with MI in both standard and restriction-adapted versions compared to CCA and ACA respectively, consistent with previous studies [4, 5]. This was presumably because we used maternal depression (a fully observed time-dependent variable) in the imputation models, which was a strong predictor of missingness [4, 21, 31, 39, 40].

The standard FCS approach imputing smoking using multinomial or ordinal logistic regression imputation failed to converge in 95–100% of the simulated datasets. Our findings agree with the results of simulation studies by Welch et al. [9] and Kalaycioglu et al. [5], which reported convergence issues in FCS, albeit of smaller proportions. Welch et al. [9], assumed that non-smokers at baseline remained non-smokers throughout, and only current- and ex-smokers transitioned between the two categories, thus converting the imputation of maternal smoking into a binary imputation. Despite this, approximately 25% of the simulated datasets did not converge with standard FCS [9]. Of note, application of the two-fold FCS in our simulation study, which reduced the number of categorical predictor variables in each univariate imputation model [30] where imputation of smoking was performed using multinomial logistic regression, still did not overcome the convergence issues. We observed similar convergence issues as seen in the simulation study with the real data.

Multinomial logistic regression faces difficulties of convergence when the imputation model includes a large number of categorical variables with rare categories and/or high collinearity. In our study, under FCS, six categorical smoking variables (one for each time point) were included in the multinomial logistic imputation model, and only a small number of ex-smokers were present in the simulated data mimicking the real cohort. Even though under the two-fold FCS algorithm only four categorical smoking variables (current and immediately adjacent time points, and smoking during pregnancy) were included in the multinomial logistic imputation model, all of these variables had a rare category leading to convergence issues.

FCS with PMM imputation produced the least biased estimates when compared to other MI methods irrespective of whether restrictions were applied. It also produced the smallest MSE across the 6 missing mechanisms, gaining precision over ACA, which performed best in terms of bias. While all other MI methods either failed to converge for all simulated datasets or resulted in large bias, PMM performed well both with and without restrictions. PMM replaces missing values with observed values [29, 41], therefore, even without restrictions, the proportion of implausible transitions imputed was low. PMM also avoids the problems arising from rounding methods related to MVNI. Slight issues of over-coverage were observed under PMM. Rodwell et al. [42] also reported issues with coverage when using PMM for imputing limited range variables, due to the matching algorithm used in Stata for PMM imputation. PMM uses three different types (0, 1 and 2) of matching to calculate a predictive distance between an observed value and a value obtained from a linear predictor, and identifies *k* observations which minimise this predictive distance. The `mi impute pmm’ command in Stata uses type 2 matching. PMM can also be implemented in R using the `mice’ package which uses type 1 matching. Type 2 matching differs from type 1 matching in that it does not adequately account for the uncertainty around the parameter of the imputation model when computing the predictive distance. A simulation study by Morris et al. [41] reported under-coverage for PMM under both type 1 and type 2 matching, with type 2 matching leading to slightly worse coverage probabilities for this reason. Therefore, the coverage probabilities may have been better when implementing PMM using the `mice’ package in R compared to the `mi impute pmm’ command in Stata.

Simulation studies by Kalaycioglu et al. [5] and De Silva et al. [4] have shown that MVNI can have very good performance when used to impute missing longitudinal data. However, the underlying assumption of multivariate normality is not plausible in our study as maternal smoking is a categorical variable. While MVNI can result in valid inferences despite the departure from multivariate normality [6, 43], adoption of a suitable rounding method to deal with non-integer imputed smoking values is required for the analysis of interest. There are number of rounding techniques available for categorical variables at a single time point [44, 45], rounding methods in the context of longitudinal data are yet to be explored [32]. We observed high biases with both MVNI approaches under different scenarios, especially without restrictions. Presumably because, indicator-PDBR uses an indicator based approach for imputation followed by projected distance-based rounding, which does not aim to preserve the marginal proportion in each category, and continuous-calibration imputes maternal smoking as a continuous variable, followed by calibration for rounding, which distorts the association between the exposure and outcome, even though it aims to preserve the marginal proportion in each category [44, 45]. Continuous-calibration resulted in substantial reductions in bias when restrictions were applied, and there were slight gains in MSE from continuous-calibration compared to indicator-PDBR, which agrees with the findings of Galati et al. [45]. It should, however, be noted that continuous-calibration was originally proposed for ordinal variables [44], while maternal smoking is technically a nominal variable. Indicator-PDBR also faced some convergence issues, presumably because it uses an indicator-based approach for imputation [44].

The three-stage restriction procedure employed in our study is an extension of the semi-deterministic approach used by Welch et al. [9], where they simplified the imputation to ex- and current-smokers as discussed previously. We observed moderate to substantial reductions in bias for PMM and continuous-calibration, and fewer convergence issues for indicator-PDBR, when restrictions were applied. However, when restrictions were applied, we observed that the empirical standard errors either slightly increased or remained the same compared with the standard implementation of MI. The MSE was greatly influenced by the empirical standard error due to its relatively large magnitude compared with absolute bias, therefore, even in scenarios which showed substantial improvements in bias, little or no change in empirical standard errors resulted in no changes in MSE, when restrictions were applied.

There is currently limited guidance on the imputation of missing values in time-dependent categorical variables even without restrictions. With standard FCS often facing convergence issues in the presence of categorical variables with rare categories, and unsatisfactory rounding methods for MVNI, this area warrants further research. Enders et al. [32] suggested using a joint imputation procedure with latent variable formulation for categorical variables, available in the MLwiN software [46]. The ‘jomo’ package in R is designed for multilevel joint modelling MI [47], but to date has not been widely adopted. Our study was limited to currently available methods in the Stata statistical software and multilevel MI methods such as ‘jomo’ are currently not available in Stata. Additionally, further research is required to examine how to implement restrictions within these multilevel imputation methods, and this was beyond the scope of this study.

Our simulation study was designed based on the LSAC infant cohort to assess the performance of MI methods in a realistic setting [4, 21, 36]. We also provide a case study for an empirical illustration of what we observed in the simulation study. This simulation study was designed based on a single cohort, and the performance of the methods may vary with changes in various factors including, magnitude and structure of the correlations between the repeated measurements, and magnitudes of the parameters used in the simulation models [21]. Therefore, caution is required when generalising these results.

## Conclusion

The findings from this study, which was based on a longitudinal cohort study, indicate that among the MI methods available in Stata (which are all single-level fixed-effect models), FCS with PMM, applied with restrictions, performs best in terms of bias and precision, when handling up to 65% missing values in a time-dependent categorical exposure variable with restrictions on transitioning over time. In a similar longitudinal setting, we would recommend the use of PMM within the FCS framework with a suitable procedure to implement restrictions within the imputations.

## Additional file

Additional file 1: Comprehensive details and findings of simulation study including Stata code. (DOCX 162 kb)

## Abbreviations

ACA: available case analysis
BMI: body mass index
BMIz: BMI for age z-scores
CCA: complete case analysis
continuous-calibration: imputation as a continuous variable using multivariate normal imputation with calibration
FCS: fully conditional specification
indicator-PDBR: indicator based imputation using multivariate normal imputation with projected distance-based rounding
LSAC: Longitudinal Study of Australian Children
MAR: missing at random
MCAR: missing completely at random
MI: multiple imputation
MSE: Mean square error
MVNI: multivariate normal imputation
PMM: predictive mean matching
two-fold FCS: two-fold fully conditional specification
