# Supplementary material for: Multiple imputation methods for handling missing values in a longitudinal categorical variable with restrictions on transitions over time: a simulation study
Source: BMC Med Res Methodol. 2019 Jan 10;19:14. doi: 10.1186/s12874-018-0653-0 (PMC6329074; doi:10.1186/s12874-018-0653-0)
Supplement: Supplementary file 1 — Comprehensive details and findings of simulation study including Stata code. (DOCX 162 kb) [file 12874_2018_653_MOESM1_ESM.docx]

**Additional file 1**

Table S1. Respondents by wave of data collection in the Longitudinal Study of Australian Children

| Infant cohort | Wave 1  2004 | Wave 2  2006 | Wave 3  2008 | Wave 4  2010 | Wave 5  2012 | Wave 6  2014 |
| --- | --- | --- | --- | --- | --- | --- |
| Age (years) | 0 – 1 | 2 – 3 | 4 -5 | 6 - 7 | 8 - 9 | 10 – 11 |
| Number of participants | 5107 | 4606 | 4386 | 4242 | 4085 | 3764 |
| Unit non-response |  | 10% | 14% | 17% | 20% | 26% |

# **Simulation of complete data**

The simulation study was based on the infant cohort of the Longitudinal Study of Australian Children (LSAC) (see Table S1). Data were generated for each child i= 1,..., *N*, where N=1,000 for waves j=1,…,6.

A total of 1,000 datasets were simulated, as descried below. Table S2 lists the parameter values used in the simulation process, which were chosen to mimic the LSAC data. A description of the variables used in the simulation equations is provided in Table 1 in the main text.

1. Generated maternal age at child birth (m_age_i_) from a normal distribution.
2. Generated maternal education (m_education_i_), from a logistic regression model conditional on maternal age at child birth:

$logit \left\{ \Pr\left( {m\_education}_{i}=1 \right) \right\}= \alpha_{0}+ \alpha_{1}{m\_age}_{i}$ (S1)

1. Generated family socio-economic status z-score (ses_i_), from a linear regression model conditional on maternal age at child birth and maternal education:

${ses}_{i}= \rho_{0}+ \rho_{1}{m\_age}_{i}+ \rho_{2}\left[ {m\_education}_{i}=1 \right]+ \psi_{i}$ (S2)

where $\psi_{i}$ is identically and independently distributed as; $\psi_{i}\sim N (0, \sigma_{\psi}^{2})$

1. Generated maternal depression at wave 0 (i.e. during pregnancy) (m_depression_i,0_) from a logistic regression model conditional on maternal age at child birth, maternal education and family socio-economic status z-score at baseline:

$logit \left\{ \Pr\left( {m\_depression}_{i,0}=1 \right) \right\}= \lambda_{0}+ \lambda_{1}{m\_age}_{i} + \lambda_{2}\left[ {m\_education}_{i}=1 \right]+ \lambda_{3}{ses}_{i}$ (S3)

While in reality maternal depression during pregnancy may vary across different trimesters, for simplicity we have simulated maternal depression to not vary during pregnancy.

1. Generated maternal smoking at wave 0 (i.e. during pregnancy) (m_smoking_i,0_) from a multinomial logistic regression conditional on maternal age at child birth, maternal education and family socio-economic status z-score at baseline:

$logit \left\{ \Pr\left( {m\_smoking}_{i,0}=a \right) \right\}= \eta_{0,a}+ \eta_{1,a}{m\_age}_{i} + \eta_{2,a}\left[ {m\_education}_{i}=1 \right]+\eta_{3,a}{ses}_{i}$ (S4)

where a = 0 (never-smoker – reference category), 1 (ex-smoker) and 2 (current-smoker)

1. Generated study child’s sex (sex_i_) by randomly assigning p% of respondents to be male.
2. Generated study child’s birth weight (birthweight_i_) from a linear regression model conditional on maternal age at child birth, maternal education, maternal smoking during pregnancy, maternal depression during pregnancy, study child’s sex, and family socio-economic status z-score at baseline:

${birthweight}_{i}= \gamma_{0}+ \gamma_{1}{m\_age}_{i}+ \gamma_{2}\left[ {m\_education}_{i}=1 \right]+ \gamma_{3}\left[ {m\_depression}_{i,0}=1 \right]+ \sum_{a=1}^{2} \gamma_{4,a}\left[ {m\_smoking}_{i,0}=a \right]+ \gamma_{5}\left[ {sex}_{i}=1 \right]+ \gamma_{6}{ses}_{i}+ \varphi_{i}$ (S5)

where $\varphi_{i}$ is identically and independently distributed as; $\varphi_{i}\sim N (0, \sigma_{\varphi}^{2})$ and a = 0 (never-smoker – reference category), 1 (ex-smoker) and 2 (current-smoker)

1. Generated breastfeeding (breastfed_i_) from a logistic regression model conditional on maternal age at child birth, maternal education, maternal depression during pregnancy and family socio-economic status z-score at baseline:

$logit \left\{ \Pr\left( {breastfed}_{i}=1 \right) \right\}= \iota_{0}+ \iota_{1}{m\_age}_{i} + \iota_{2}\left[ {m\_education}_{i}=1 \right]+ \iota_{3} \left[ {m\_depression}_{i,0}=1 \right]+ \iota_{4}{ses}_{i}$ (S6)

1. Generated maternal depression for waves j=1,…,6 (m_depression_i,j_) using a logistic regression model:

$logit \left\{ \Pr\left( {m\_depression}_{i,j}=1 \right) \right\}= \delta_{0}+ \delta_{1}{m\_age}_{i} {+ \delta}_{2}{[m\_education}_{i}=1] +\delta_{3}{[m\_depression}_{i,j-1}=1]+ \delta_{4}{ses}_{i}$ (S7)

1. Generated maternal smoking at waves j=1,…,6 (m_smoking_i,j_) in two stages.

- Stage 1: Generated maternal smoking for respondents who were never-smokers at the previous wave using the multinomial logistic regression model:

$logit \left\{ \Pr\left( {m\_smoking}_{i,j}=b| {m\_smoking}_{i,j-1}=0 \right) \right\}= \zeta_{0,a}+ \zeta_{1,a}{m\_age}_{i}+ \zeta_{2,a}\left[ {m\_education}_{i}= 1 \right]+ \zeta_{3,a}\left[ {m\_depression}_{i,j-1}= 1 \right]+ \zeta_{4,a}{ses}_{i}$ (S8)

where b = 0 (never-smoker – reference category), 1 (ex-smoker) and 2 (current-smoker)

- Stage 2: Generated maternal smoking for the remaining respondents (current- or ex-smoker) using the logistic regression model:

$logit \left\{ \Pr\left( {m\_smoking}_{i,j}=2| {m\_smoking}_{i,j-1}\neq0 \right) \right\}= \kappa_{0}+ \kappa_{1}{m\_age}_{i}+ \kappa_{2}\left[ {m\_education}_{i}= 1 \right]+ \kappa_{3}\left[ {m\_depression}_{i,j-1}= 1 \right]+ \kappa_{4}\left[ {m\_smoking}_{i,j-1}= 2 \right] + \kappa_{5}{ses}_{i}$ (S9)

where 1 (ex-smoker – reference category) and 2 (current-smoker)

1. Generated study child’s age at wave 1 (scage_i,1_) from a normal distribution, ${scage}_{i,1} \sim N (Mean, {Standard deviation}^{2})$. The study child’s age values for the succeeding waves were generated by adding the time gap between the waves to the age of the preceding wave (24 months).

${scage}_{i,j}= 24+ {scage}_{i,j-1}$ (S10)

1. Generated body mass index (BMI) for age z-scores (BMIz_i,j_) for waves j=2,…,6 using a linear mixed-effects model:

${BMIz}_{i,j}=\left( \theta_{0}+b_{0i} \right)+{{\sum_{a=1}^{2} \theta_{1,a}\left[ {m\_smoking}_{i,j-1}=a \right]+\theta}_{2}{scage}_{ij}+\theta_{3}\left[ {breastfed}_{i}=1 \right]+ \theta_{4}{m\_age}_{i}+ \theta}_{5}\left[ {m\_education}_{i}=1 \right]+ \theta_{6}{birthweight}_{i}+ \theta_{7}\left[ {sex}_{i}=1 \right]+\theta_{8}{ses}_{i} +\varepsilon_{ij}$ (S11)

where $\varepsilon_{\mathrm{ij}}$ is identically and independently distributed $\sim N (0, \sigma_{\varepsilon}^{2})$, a = 0 (never-smoker – reference category), 1 (ex-smoker) and 2 (current-smoker), and values for the random intercept b_0i_ were drawn from $\sim N (0, \sigma_{\varphi}^{2})$.

Table S2: Details of the parameters used in the data generation models

| Variable Generated |  | Explanatory Variable | | | | Parameter Value |
| --- | --- | --- | --- | --- | --- | --- |
| Maternal age at child birth (years) |  |  | | | Mean | 31 |
|  |  |  | | | Standard deviation | 5 |
|  |  |  | | |  |  |
| Maternal education |  | Constant | | | $\alpha_{0}$ | -3.1 |
|  |  | Maternal age, years | | | $\alpha_{1}$ | 0.1 |
|  |  |  | | |  |  |
| Family socio-economic status (SES) z-score |  | Constant  Maternal age, years  Maternal education_Completed_ | | | $\rho_{0}$  $\rho_{1}$  $\rho_{2}$ | -1.3  0.03  1.1 |
|  |  | Error term | | | Mean  Standard deviation $\sigma_{\psi}$ | 0  0.7 |
|  |  |  | | |  |  |
| Maternal depression at wave 0 (i.e. during pregnancy) |  | Constant  Maternal age, years | | | $\lambda_{0}$  $\lambda_{1}$ | -2.3  0.03 |
|  |  | Maternal education_Completed_ | | | $\lambda_{2}$ | -0.4 |
|  |  | Family SES z-score | | | $\lambda_{3}$ | -0.3 |
|  |  |  |  |  |  |  |
|  |  |  | |  | |  |
| Maternal smoking at wave 0 (i.e. during pregnancy) |  |  | |  | |  |
| *Ex-smoker* |  | Constant | | $\eta_{01}$ | | -1.6 |
|  |  | Maternal age, years | | $\eta_{11}$ | | -0.05 |
|  |  | Maternal education_Completed_ | | $\eta_{21}$ | | -0.5 |
|  |  | Family SES z-score | | $\eta_{31}$ | | -0.3 |
|  |  |  | |  | |  |
| *Current-smoker* |  | Constant | | $\eta_{02}$ | | 0.5 |
|  |  | Maternal age, years | | $\eta_{12}$ | | -0.05 |
|  |  | Maternal education_Completed_ | | $\eta_{22}$ | | -0.6 |
|  |  | Family SES z-score | | $\eta_{32}$ | | -0.4 |
|  |  |  | |  | |  |
| Study child’s sex |  | Male proportion | | p | | 0.5 |
|  |  |  | |  | |  |
| Study child’s birth weight, kg |  | Constant | | $\gamma_{0}$ | | 3.2 |
|  |  | Maternal age, years | | $\gamma_{1}$ | | -0.001 |
|  |  | Maternal education_Completed_ | | $\gamma_{2}$ | | 0.2 |
|  |  | Maternal depression at wave 0_Yes_ | | $\gamma_{3}$ | | -0.1 |
|  |  | Maternal smoking at wave 0_Never_  Maternal smoking at wave 0_Ex_  Maternal smoking at wave 0_Current_ | | $\gamma_{40}$  $\gamma_{41}$  $\gamma_{42}$ | | 0  -0.18  -0.20 |
|  |  | Study child’s sex_Male_ | | $\gamma_{5}$ | | 0.13 |
|  |  | Family SES z-score | | $\gamma_{6}$ | | 0.05 |
|  |  | Error term | | Mean | | 0 |
|  |  |  |  | Standard deviation $\sigma_{\varphi}$ | | 0.6 |
|  |  |  | |  | |  |
| Breastfeeding patterns |  | Constant | | $\iota_{0}$ | | -1.5 |
|  |  | Maternal age, years | | $\iota_{1}$ | | 0.05 |
|  |  | Maternal education_Completed_ | | $\iota_{2}$ | | 0.5 |
|  |  | Maternal depression at wave 0_Yes_ | | $\iota_{3}$ | | -0.5 |
|  |  | Family SES z-score | | $\iota_{4}$ | | 0.3 |
|  |  |  | |  | |  |
| Maternal depression at waves j=1,…,6 |  | Constant  Maternal age, years | | $\delta_{0}$  $\delta_{1}$ | | -1.8  0.01 |
|  |  | Maternal education_Completed_ | | $\delta_{2}$ | | -0.4 |
|  |  | Maternal depression at previous wave_Yes_ | | $\delta_{3}$ | | 1.0 |
|  |  | Family SES z-score | | $\delta_{4}$ | | -0.3 |
|  |  |  | |  | |  |
| Maternal smoking at waves j=1,…,6 |  |  | |  | |  |
|  |  |  | |  | |  |
| *Stage 1: Respondents who were never-smokers at the previous wave* | | | |  | |  |
| Ex-smoker |  | Constant  Maternal age, years | | $\zeta_{01}$  $\zeta_{11}$ | | -3.0  -0.04 |
|  |  | Maternal education_Completed_ | | $\zeta_{21}$ | | -0.5 |
|  |  | Maternal depression at previous wave_Yes_ | | $\zeta_{31}$ | | 1.0 |
|  |  | Family SES z-score | | $\zeta_{41}$ | | -0.3 |
|  |  |  | |  | |  |
| Current-smoker |  | Constant  Maternal age, years | | $\zeta_{02}$  $\zeta_{12}$ | | -2.5  -0.05 |
|  |  | Maternal education_Completed_ | | $\zeta_{22}$ | | -0.6 |
|  |  | Maternal depression at previous wave_Yes_ | | $\zeta_{32}$ | | 1.2 |
|  |  | Family SES z-score | | $\zeta_{42}$ | | -0.4 |
|  |  |  | |  | |  |
| *Stage 2: Respondents who were current- or ex-smokers at the previous wave* | | | | | | |
| Current-smoker |  | Constant  Maternal age, years | | $\kappa_{0}$  $\kappa_{1}$ | | 1.1  -0.05 |
|  |  | Maternal education_Completed_ | | $\kappa_{2}$ | | -0.5 |
|  |  | Maternal depression at previous wave_Yes_ | | $\kappa_{3}$ | | 1.2 |
|  |  | Maternal smoking at previous wave_Current_ | | $\kappa_{4}$ | | 1.0 |
|  |  | Family SES z-score | | $\kappa_{5}$ | | -0.4 |
|  |  |  | |  | |  |
| Study child’s age at wave 1 (months) |  |  | | Mean | | 9 |
|  |  |  | | Standard deviation | | 2.6 |
|  |  |  | |  | |  |
| BMI for age z-scores at waves |  | | Constant | | $\theta_{0}$ | -0.1 |
| j=1,…,6 |  | | Maternal smoking at previous wave_Never_ | | $\theta_{10}$ | 0 |
|  |  | | Maternal smoking at previous wave_Ex_ | | $\theta_{11}$ | 0.10 |
|  |  | | Maternal smoking at previous wave_Current_ | | $\theta_{12}$ | 0.15 |
|  |  | | Study child’s age, months | | $\theta_{2}$ | -0.005 |
|  |  | | Breastfeeding patterns_Never_ | | $\theta_{30}$ | 0 |
|  |  | | Breastfeeding patterns | | $\theta_{3}$ | -0.13 |
|  |  | | Maternal age, years | | $\theta_{4}$ | 0.01 |
|  |  | | Maternal education_Completed_ | | $\theta_{5}$ | -0.02 |
|  |  | | Birth weight, kg | | $\theta_{6}$ | -0.01 |
|  |  | | Study child’s sex_Female_ | | $\theta_{7}$ | -0.03 |
|  |  | | Family SES z-score | | $\theta_{8}$ | -0.08 |
|  |  | | Error term | | Mean | 0 |
|  |  | |  | | Standard deviation $\sigma_{\varepsilon}$ | 0.7 |
|  |  | | Random Effects   - Standard deviation | | $\sigma_{\varphi}$ | 0.8 |
|  |  | |  | |  |  |

Abbreviations: BMI, body mass index; kg, kilograms.

**Complete-data analysis**

We conducted a complete-data analysis before introducing missingness and obtained the parameter estimates, 0.0998 and 0.1495 for the association between childhood obesity and exposure to maternal smoking for ex-smokers and current-smokers respectively. The true values (i.e. values used to simulate the association between childhood obesity and exposure to maternal smoking, Equation S11 in Additional File 1) were 0.1 and 0.15 for ex-smokers and current-smokers respectively, which provided a check that the data were simulated correctly.

**The d-separation criterion**

The d-separation criterion, determines the statistical independence of two variables given a set of related other variables [1]. This was used to identify a set of variables that could be used to predict the missingness in maternal smoking to give a missing at random (MAR) mechanism. For the MAR assumption to hold, the probability of missingness should be independent of the variable with missing values conditional on the observed data [2].

The time-dependent outcome variable BMI for age z-scores (BMIz) and the time-dependent auxiliary variable maternal depression were selected as predictors of missingness. As per the d-separation criterion, for the MAR assumption to hold, the indicator of missingness in maternal smoking at wave j (R_j_, maternal smoking measures at wave j are missing if R_j_ =1) should be statistically independent of maternal smoking measured at wave j (m_smoking_j_), conditional on both maternal depression at wave j (m_depression_j_) and BMI for age z-scores measured at the subsequent wave (BMIz_j+1_) (Equation S12).

${m\_smoking}_{j}\perp R_{j} | {m\_depression}_{j}, \mathrm{BMIz}_{j+1}$ (S12)

To prove that the MAR assumption holds for the chosen predictors of missingness we need to prove that the variables m_smoking_j_ and R_j_ are d-separated, when conditioned on both m_depression_j_ and BMIz_j+1_. The associations shown in Figure 1b in the main text are used to show that the d-separation holds under the chosen predictors of missingness.

We consider the association between m_smoking_j_ and R_j_ separately for both of the potential predictors of missingness. The association between m_smoking_j_ and R_j_ through maternal depression as shown in Figure 1b in the main text is; m_smoking_j_🡨 m_depression_j-1_ 🡪 m_depression_j_🡪 R_j_. The path between the two variables is open, resulting in the two variables m_smoking_j_ and R_j_ to be d-connected. However if we condition on m_depression_j_, the path becomes blocked [1]. Similarly m_smoking_j_ and R_j_ are d-connected through the open path containing BMIz_j+1_; m_smoking_j_ 🡪 BMIz_j+1_🡪 R_j_. In order to block the path, we need to condition on BMIz_j+1_. Therefore, conditioning on both m_depression_j_ and BMIz_j+1_ leads to the d-separation of m_smoking_j_ and R_j_, thus satisfying the MAR assumption.

# Therefore, under MAR, we assumed that the probability of missingness in maternal smoking at waves 1 to 5 followed a logistic regression model dependent on maternal depression and BMI for age z-scores (see Figure 1b in the main text).

See Equations 5 and 6 in the main text for the two logistic regression models used to generate missing values in maternal smoking. The required proportions of missing maternal smoking values at each wave were obtained by choosing appropriate intercepts for the logistic regression models, $\nu_{0,j}$ (Equation 5 in the main text) and $\omega_{0,j}$ (Equation 6 in the main text) by iteration.


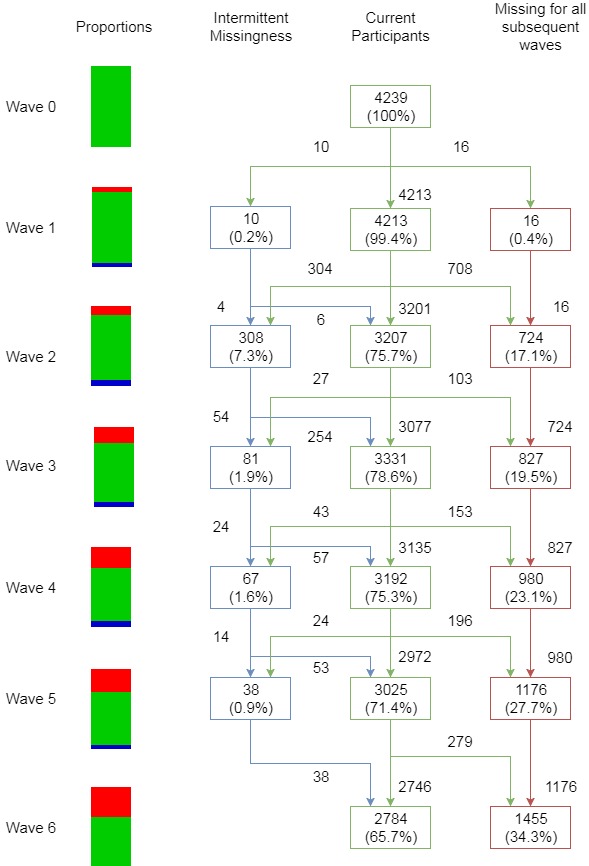
 Figure S1. Missing data patterns for maternal smoking in the infant cohort of the Longitudinal Study of Australian Children. Percentage of respondents who had all maternal smoking measurements missing after each wave is presented as missing for all subsequent waves, and those who returned at a later wave are presented as intermittent missingness.

# **Rounding methods for multivariate normal imputation**

As described in the main text, due to the assumption of joint multivariate normality, missing values imputed under multivariate normal imputation (MVNI) could be non-integer values, and therefore rounding is required to re-categorise the imputed values into its original form. We compared the results of two methods in our simulation study:

- Indicator based imputation using MVNI with projected distance-based rounding (indicator-PDBR):

indicator-PDBR is based on the imputation of indicator variables corresponding to the categorical variable maternal smoking using MVNI followed by projected distance-based rounding (see Figure S2) [3]. We chose indicator-PDBR as it is one of the few methods available for nominal variables, and it has been shown that indicator-PDBR preserves the association between the outcome and exposure variables (i.e. this could result in less biased parameter estimates) compared to other rounding methods [4, 5].

**Step 1: Create indicator variables corresponding to the incomplete variable with k+1 categories**

$$I_{ijk}= \left\{ \begin{aligned} 1 if {m\_smoking}_{ij}=k \\ 0 if {m\_smoking}_{ij}\neq k \\ missing if {m\_smoking}_{ij}=missing \end{aligned} \right.i=1,\ldots,1,000 j=1,\ldots,5 k=1,2$$

**Step 2: Impute missing values in the indicator variables using multivariate normal imputation** **(MVNI)**

**Step 3: Create indicator variables for the reference category for the imputed observations**

$$I_{ij0}= 1-I_{ij1}- I_{ij2} i=1,\ldots,1,000 j=1,\ldots,5$$

**Step 4: Choose the largest indicator**

$${maximum\_indicator}_{ij}= maximum (I_{ij0} I_{ij1} I_{ij2} ) i=1,\ldots,1,000 j=1,\ldots,5$$

**Step 5: Replace missing values by the category corresponding to the maximum indicator**

$${m\_smoking}_{ij}= a if {maximum\_indicator}_{ij}=I_{ija} i=1,\ldots,1,000 j=1,\ldots,5, a=0,\ldots,2$$

Figure S2. Stages for indicator-based imputation using multivariate normal imputation with projected distance-based rounding; I_ijk_, Indicator variable for the respondent i, at wave j corresponding to category k of maternal smoking (m_smoking).

- **Imputation as a continuous variable using MVNI with calibration (continuous-calibration):**

The categorical variable maternal smoking is imputed as a continuous variable using MVNI, followed by calibration in a two-stage approach (see Figure S3). continuous-calibration recommended for the imputation of missing values in ordinal variables [6, 7]. This method focuses on preserving marginal proportions observed in the complete data (i.e. re-categorises imputed values so that the overall proportions observed for maternal smoking categories in the complete data remains unchanged) [4].

**Stage 1**

- Calculate the proportion of respondents belonging to the k^th^ category for maternal smoking at wave j (prop_jk_; j=1,…,5 and k=0,…,2)
- Create a duplicate entry for each respondent and assign all values of maternal smoking at waves 1 to 5, to missing in the duplicated dataset
- Impute missing values in maternal smoking at waves 1 to 5 in the combined dataset (i.e. both original and duplicate) using multivariate normal imputation (MVNI). The nominal variables are imputed under the assumption of multivariate normality.
- Retain imputed values in the duplicated dataset and categorise them (as 0 never, 1 ex or 2 current-smoker) based on the observed proportions (prop_jk_)
- Calculate cut off points based on the imputed values (cut_jm_; j=1,…,5 and m=1,2)

**Stage 2**

- Impute missing values in maternal smoking at waves 1 to 5 in the original dataset using MVNI
- Re-categorise the imputed values into the original categories based on the cut points computed in stage 1

$${m\_smoking}_{ij}= \left\{ \begin{aligned} 0 if {m\_smoking}_{ij}\leq{cut}_{j1} \\ 1 if {{cut}_{j1}<m\_smoking}_{ij}\leq{cut}_{j2} \\ 2 if {m\_smoking}_{ij}>{cut}_{j2} \end{aligned} \right. i=1,\ldots,1,000 j=1,\ldots,5$$

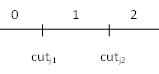


Figure S3. Stages for continuous imputation using multivariate normal imputation with calibration method for rounding; m_smoking_ij_, maternal smoking for the respondent i, at wave j.

**The two-fold fully conditional specification (two-fold FCS) algorithm**

The following settings were used to impute missing data using the two-fold FCS algorithm in Stata:

- Number of imputations $(m)$: 35 or 65 depending on the percentage of missing data [8]
- Time window width $(width)$: 1 (i.e. only use information from specific and immediately adjacent time points for imputation) [9]
- Number of among-time iterations $(ba)$: 20 iterations [9, 10]
- Number of within-time iterations $(bw)$: 5 iterations [9, 10]

**Case study analysis**

Maternal smoking from pregnancy (wave 0) up to waves 6 was recorded as a binary variable; “*Is the mother a current-smoker or not?*”. For the purpose of this case study we generated a new categorical variable with three levels (never-smoker, ex-smoker and current-smoker) from waves 1 to 6, using the binary maternal smoking variable as follows:

- - - - A respondent observed as a smoker at a specific wave was categorised as a current-smoker at that particular wave.
- A respondent observed as a non-smoker at a specific wave and all other previous waves was categorised as a never-smoker at that particular wave.
- A respondent observed as a non-smoker at a specific wave, however was observed as a smoker at any of the previous waves was categorised as an ex-smoker at that particular wave.
- In the scenario where a respondent was observed as a non-smoker at a specific wave and one or more of the previous waves, however has values missing in between was assigned as missing at that particular wave.

This time-dependent exposure had restrictions on transitions over time, where a respondent recorded as a current or ex-smoker at a specific wave could not transition into a never-smoker at a later wave. In the case study example, none of the respondents transitioned from a never-smoker at a specific wave to an ex-smoker at a subsequent wave. The number of women who transitioned from a never-smoker at wave $j-1$ to a current-smoker at wave $j$, for $j=1,\ldots,5$ were 283, 72, 39, 30 and 26, respectively.

Table S3. Estimated mean difference in body mass index for age z-scores for ex-smokers and current-smokers compared to never-smokers for the case study analysis obtained with a random intercept linear mixed-effects model using different methods for handling missing data in maternal smoking

| Missing Data Method^a^ | Ex-smoker | | | Current-smoker | | |
| --- | --- | --- | --- | --- | --- | --- |
|  | Parameter (95% CI) | SE | p-value | Parameter (95% CI) | SE | p-value |
| CCA | 0.216 (0.115, 0.318) | 0.052 | <0.001 | 0.192 (0.103, 0.280) | 0.045 | <0.001 |
| ACA | 0.171 (0.090, 0.251) | 0.041 | <0.001 | 0.175 (0.109, 0.241) | 0.034 | <0.001 |
| indicator-PDBR | 0.183 (0.087, 0.279) | 0.049 | <0.001 | 0.179 (0.097, 0.262) | 0.042 | <0.001 |
| continuous-calibration | 0.177 (0.083, 0.272) | 0.048 | <0.001 | 0.179 (0.097, 0.261) | 0.042 | <0.001 |
| continuous-calibration-R | 0.182 (0.085, 0.279) | 0.049 | <0.001 | 0.179 (0.096, 0.262) | 0.042 | <0.001 |
| PMM-5^b^ | 0.186 (0.088, 0.284) | 0.050 | <0.001 | 0.181 (0.099, 0.264) | 0.042 | <0.001 |
| PMM-5-R^b^ | 0.190 (0.094, 0.286) | 0.049 | <0.001 | 0.185 (0.102, 0.268) | 0.042 | <0.001 |

Abbreviations: ACA, available case analysis; CCA, complete case analysis; CI, confidence interval; continuous-calibration, imputation as a continuous variable using multivariate normal imputation with calibration; continuous-calibration-R, imputation as a continuous variable using multivariate normal imputation with calibration and restrictions; indicator-PDBR, indicator based imputation using multivariate normal imputation with projected distance-based rounding; PMM-5, predictive mean matching with 5 nearest neighbours; PMM-5-R, predictive mean matching with 5 nearest observations and restrictions; SE, standard error.

^a^ Methods in which the imputation models failed to converge are not shown.

^b^Minimal differences were observed between the results of predictive mean matching with 5 and 10 nearest observations. Therefore, only the results for this method with 5 nearest observations are presented.

Table S4. Performance of the various methods for handling 45% missingness in maternal smoking for the parameter estimate^b^ of current-smokers relative to never-smokers for the simulation study

| Performance Measure | Missing Data Method^c^ | | | | | | | | | |
| --- | --- | --- | --- | --- | --- | --- | --- | --- | --- | --- |
|  | CCA | ACA | MVNI | | | | PMM^d^ | | | |
|  |  |  | indicator-PDBR | | continuous-calibration | |  | | | |
|  |  |  | Without | With | Without | With | Without | With | | |
|  |  |  | Restrictions | Restrictions | Restrictions | Restrictions | Restrictions | Restrictions | | |
| **MCAR** |  | |  | |  | |  | |  |  |
| Absolute Bias^a^ | 0.000 | 0.001 | 0.010 | 0.008 | 0.011 | 0.007 | 0.008 | 0.007 | | |
| Relative Bias (%) | 0.045 | 0.642 | 6.418 | 5.336 | 7.282 | 4.949 | 5.533 | 4.598 | | |
| Empirical SE | 0.072 | 0.056 | 0.052 | 0.053 | 0.052 | 0.052 | 0.053 | 0.053 | | |
| Model-based SE | 0.071 | 0.057 | 0.056 | 0.056 | 0.056 | 0.056 | 0.056 | 0.056 | | |
| Coverage (%) | 94.9 | 95.5 | 95.6 | 95.3 | 95.5 | 95.4 | 95.4 | 95.0 | | |
| MSE | 0.005 | 0.003 | 0.003 | 0.003 | 0.003 | 0.003 | 0.003 | 0.003 | | |
| **MAR (weak)** |  |  |  |  |  |  |  |  | | |
| Absolute Bias^a^ | 0.009 | 0.002 | 0.013 | 0.013 | 0.012 | 0.007 | 0.009 | 0.006 | | |
| Relative Bias (%) | 5.953 | 1.101 | 8.577 | 8.703 | 8.078 | 4.638 | 5.880 | 4.053 | | |
| Empirical SE | 0.070 | 0.055 | 0.052 | 0.052 | 0.051 | 0.052 | 0.052 | 0.053 | | |
| Model-based SE | 0.072 | 0.057 | 0.056 | 0.056 | 0.056 | 0.056 | 0.056 | 0.056 | | |
| Coverage (%) | 95.2 | 95.9 | 96.0 | 95.7 | 96.1 | 95.7 | 96.1 | 95.4 | | |
| MSE | 0.005 | 0.003 | 0.003 | 0.003 | 0.003 | 0.003 | 0.003 | 0.003 | | |
| **MAR (strong)** |  |  |  |  |  |  |  |  | | |
| Absolute Bias^a^ | 0.018 | 0.006 | 0.018 | 0.019 | 0.015 | 0.008 | 0.011 | 0.006 | | |
| Relative Bias (%) | 12.060 | 4.157 | 11.807 | 12.967 | 9.682 | 5.324 | 7.126 | 4.000 | | |
| Empirical SE | 0.067 | 0.055 | 0.052 | 0.052 | 0.051 | 0.052 | 0.052 | 0.052 | | |
| Model-based SE | 0.070 | 0.057 | 0.056 | 0.056 | 0.057 | 0.056 | 0.057 | 0.057 | | |
| Coverage (%) | 96.0 | 95.8 | 96.1 | 95.4 | 96.6 | 96.6 | 96.4 | 96.4 | | |
| MSE | 0.005 | 0.003 | 0.003 | 0.003 | 0.003 | 0.003 | 0.003 | 0.003 | | |

Abbreviations: ACA, available case analysis; CCA, complete case analysis; continuous-calibration, imputation as a continuous variable using multivariate normal imputation with calibration; Empirical SE, empirical standard error; indicator-PDBR, indicator based imputation using multivariate normal imputation with projected distance-based rounding; MAR, missing at random; MCAR, missing completely at random; Model-based SE, model based standard error; MVNI, multivariate normal imputation; PMM, predictive mean matching; MSE, mean square error.

^a^Monte Carlo standard error did not exceed 0.003.

^b^True value for the mean difference in body mass index for age z-scores between current-smokers and never-smokers is 0.15.

^c^ Methods in which the imputation models failed to converge are not shown.

^d^Minimal differences were observed between the results of predictive mean matching with 5 and 10 nearest observations. Therefore, only the results for this method with 5 nearest observations are presented.

Table S5. Performance of the various methods for handling 65% missingness in maternal smoking for the parameter estimate^b^ for current-smokers relative to never-smokers for the simulation study

| Performance Measure | Missing Data Method^c^ | | | | | | | | | | | | | |
| --- | --- | --- | --- | --- | --- | --- | --- | --- | --- | --- | --- | --- | --- | --- |
|  | CCA | ACA | MVNI | | | | | | PMM^d^ | | | | | |
|  |  |  | indicator-PDBR | | | continuous-calibration | | |  | | | | | |
|  |  |  | Without | | With | Without | | With | Without | | With | | | |
|  |  |  | Restrictions^e^ | | Restrictions^f^ | Restrictions | | Restrictions | Restrictions | | Restrictions | | | |
| **MCAR** |  | |  | | |  | | |  | | |  |  |  |
| Absolute Bias^a^ | 0.002 | 0.001 | 0.016 | 0.014 | | 0.018 | 0.013 | | 0.014 | 0.011 | | | |  |
| Relative Bias (%) | 1.549 | 0.589 | 10.938 | 9.102 | | 11.960 | 8.444 | | 9.157 | 7.656 | | | |  |
| Empirical SE | 0.091 | 0.060 | 0.052 | 0.053 | | 0.053 | 0.054 | | 0.054 | 0.055 | | | |  |
| Model-based SE | 0.089 | 0.061 | 0.057 | 0.058 | | 0.058 | 0.058 | | 0.058 | 0.058 | | | |  |
| Coverage (%) | 94.1 | 95.7 | 96.2 | 96.2 | | 95.4 | 95.8 | | 96.4 | 96.3 | | | |  |
| MSE | 0.008 | 0.004 | 0.003 | 0.003 | | 0.003 | 0.003 | | 0.003 | 0.003 | | | |  |
| **MAR (weak)** |  |  |  |  | |  |  | |  |  | | | |  |
| Absolute Bias^a^ | 0.011 | 0.002 | 0.021 | 0.020 | | 0.019 | 0.011 | | 0.014 | 0.009 | | | |  |
| Relative Bias (%) | 7.196 | 1.560 | 13.833 | 13.642 | | 12.593 | 7.246 | | 9.168 | 6.265 | | | |  |
| Empirical SE | 0.085 | 0.059 | 0.053 | 0.053 | | 0.052 | 0.054 | | 0.053 | 0.054 | | | |  |
| Model-based SE | 0.086 | 0.060 | 0.058 | 0.058 | | 0.058 | 0.058 | | 0.058 | 0.058 | | | |  |
| Coverage (%) | 95.5 | 95.3 | 95.0 | 95.1 | | 95.7 | 96.1 | | 96.8 | 96.3 | | | |  |
| MSE | 0.007 | 0.003 | 0.003 | 0.003 | | 0.003 | 0.003 | | 0.003 | 0.003 | | | |  |
| **MAR (strong)** |  |  |  |  | |  |  | |  |  | | | |  |
| Absolute Bias^a^ | 0.024 | 0.010 | 0.030 | 0.031 | | 0.025 | 0.014 | | 0.018 | 0.011 | | | |  |
| Relative Bias (%) | 16.304 | 6.623 | 20.236 | 20.883 | | 16.650 | 9.154 | | 11.788 | 7.103 | | | |  |
| Empirical SE | 0.084 | 0.058 | 0.051 | 0.053 | | 0.053 | 0.054 | | 0.052 | 0.054 | | | |  |
| Model-based SE | 0.085 | 0.060 | 0.058 | 0.058 | | 0.059 | 0.059 | | 0.059 | 0.059 | | | |  |
| Coverage (%) | 94.5 | 95.6 | 94.2 | 93.9 | | 95.4 | 96.7 | | 96.2 | 97.1 | | | |  |
| MSE | 0.008 | 0.003 | 0.004 | 0.004 | | 0.003 | 0.003 | | 0.003 | 0.003 | | | |  |

Abbreviations: ACA, available case analysis; CCA, complete case analysis; continuous-calibration, imputation as a continuous variable using multivariate normal imputation with calibration; Empirical SE, empirical standard error; indicator-PDBR, indicator based imputation using multivariate normal imputation with projected distance-based rounding; MAR, missing at random; MCAR, missing completely at random; Model-based SE, model based standard error; MVNI, multivariate normal imputation; PMM, predictive mean matching; MSE, mean square error.

^a^Monte Carlo standard error did not exceed 0.003.

^b^True value for the mean difference in body mass index for age z-scores between current-smokers and never-smokers is 0.15.

^c^ Methods in which the imputation models failed to converge are not shown.

^d^Minimal differences were observed between the results of predictive mean matching with 5 and 10 nearest observations. Therefore, only the results for this method with 5 nearest observations are presented.

^e^0.2%, 0.3%, and 0.3% of the datasets failed to converge when using indicator-PDBR without restrictions under MCAR, MAR (weak), and MAR (strong) scenarios respectively.

^f^0.1% of the datasets failed to converge when using indicator-PDBR with restrictions under the MAR (strong) scenario.

Table S6. performance of the various methods for handling 45% missingness in maternal smoking for the parameter estimate^b^ for ex-smokers relative to never-smokers for the simulation study

| Performance Measure | Missing Data Method^c^ | | | | | | | | | |
| --- | --- | --- | --- | --- | --- | --- | --- | --- | --- | --- |
|  | CCA | ACA | MVNI | | | | PMM^d^ | | | |
|  |  |  | indicator-PDBR | | continuous-calibration | |  | | | |
|  |  |  | Without | With | Without | With | Without | With | | |
|  |  |  | Restrictions | Restrictions | Restrictions | Restrictions | Restrictions | Restrictions | | |
| **MCAR** |  | |  | |  | |  | |  |  |
| Absolute Bias^a^ | 0.001 | 0.000 | 0.004 | 0.004 | 0.011 | 0.006 | 0.003 | 0.004 | | |
| Relative Bias (%) | 0.764 | 0.146 | 4.457 | 4.243 | 11.123 | 5.766 | 2.983 | 3.512 | | |
| Empirical SE | 0.076 | 0.061 | 0.057 | 0.057 | 0.051 | 0.054 | 0.055 | 0.056 | | |
| Model-based SE | 0.074 | 0.060 | 0.058 | 0.058 | 0.058 | 0.058 | 0.058 | 0.058 | | |
| Coverage (%) | 94.6 | 94.5 | 95.4 | 95.0 | 96.9 | 96.3 | 96.3 | 95.3 | | |
| MSE | 0.006 | 0.004 | 0.003 | 0.003 | 0.003 | 0.003 | 0.003 | 0.003 | | |
| **MAR (weak)** |  |  |  |  |  |  |  |  | | |
| Absolute Bias^a^ | 0.009 | 0.001 | 0.008 | 0.006 | 0.019 | 0.007 | 0.002 | 0.000 | | |
| Relative Bias (%) | 9.120 | 0.576 | 7.993 | 5.986 | 18.724 | 6.998 | 2.439 | 0.312 | | |
| Empirical SE | 0.075 | 0.061 | 0.056 | 0.057 | 0.050 | 0.053 | 0.054 | 0.055 | | |
| Model-based SE | 0.074 | 0.060 | 0.059 | 0.058 | 0.058 | 0.058 | 0.059 | 0.059 | | |
| Coverage (%) | 95.0 | 95.2 | 96.4 | 95.9 | 97.5 | 97.2 | 97.0 | 96.2 | | |
| MSE | 0.006 | 0.004 | 0.003 | 0.003 | 0.003 | 0.003 | 0.003 | 0.003 | | |
| **MAR (strong)** |  |  |  |  |  |  |  |  | | |
| Absolute Bias^a^ | 0.016 | 0.005 | 0.013 | 0.010 | 0.027 | 0.009 | 0.002 | 0.003 | | |
| Relative Bias (%) | 15.736 | 4.558 | 13.068 | 9.648 | 27.284 | 9.374 | 2.233 | 2.922 | | |
| Empirical SE | 0.072 | 0.060 | 0.056 | 0.057 | 0.049 | 0.053 | 0.053 | 0.055 | | |
| Model-based SE | 0.073 | 0.059 | 0.059 | 0.059 | 0.059 | 0.059 | 0.059 | 0.059 | | |
| Coverage (%) | 95.2 | 94.7 | 95.9 | 95.4 | 96.2 | 96.7 | 97.0 | 95.9 | | |
| MSE | 0.005 | 0.004 | 0.003 | 0.003 | 0.003 | 0.003 | 0.003 | 0.003 | | |

Abbreviations: ACA, available case analysis; CCA, complete case analysis; continuous-calibration, imputation as a continuous variable using multivariate normal imputation with calibration; Empirical SE, empirical standard error; indicator-PDBR, indicator based imputation using multivariate normal imputation with projected distance-based rounding; MAR, missing at random; MCAR, missing completely at random; Model-based SE, model based standard error; MVNI, multivariate normal imputation; PMM, predictive mean matching; MSE, mean square error.

^a^Monte Carlo standard error did not exceed 0.003.

^b^True value for the mean difference in body mass index for age z-scores between ex-smokers and never-smokers is 0.10.

^c^ Methods in which the imputation models failed to converge are not shown.

^d^Minimal differences were observed between the results of predictive mean matching with 5 and 10 nearest observations. Therefore, only the results for this method with 5 nearest observations are presented.

Table S7. Performance of the various methods for handling 65% missingness in maternal smoking for the parameter estimate^b^ for ex-smokers relative to never-smokers for the simulation study

| Performance Measure | Missing Data Method^c^ | | | | | | | | | | | | | |
| --- | --- | --- | --- | --- | --- | --- | --- | --- | --- | --- | --- | --- | --- | --- |
|  | CCA | ACA | MVNI | | | | | | PMM^d^ | | | | | |
|  |  |  | indicator-PDBR | | | continuous-calibration | | |  | | | | | |
|  |  |  | Without | | With | Without | | With | Without | | With | | | |
|  |  |  | Restrictions^e^ | | Restrictions^f^ | Restrictions | | Restrictions | Restrictions | | Restrictions | | | |
| **MCAR** |  | |  | | |  | | |  | | |  |  |  |
| Absolute Bias^a^ | 0.002 | 0.000 | 0.008 | 0.008 | | 0.019 | 0.011 | | 0.006 | 0.006 | | | |  |
| Relative Bias (%) | 2.130 | 0.401 | 8.494 | 7.818 | | 19.014 | 10.556 | | 6.217 | 6.239 | | | |  |
| Empirical SE | 0.096 | 0.065 | 0.056 | 0.057 | | 0.046 | 0.050 | | 0.052 | 0.055 | | | |  |
| Model-based SE | 0.092 | 0.064 | 0.061 | 0.060 | | 0.059 | 0.060 | | 0.060 | 0.060 | | | |  |
| Coverage (%) | 94.4 | 93.2 | 96.2 | 95.6 | | 97.8 | 97.4 | | 97.0 | 96.8 | | | |  |
| MSE | 0.009 | 0.004 | 0.003 | 0.003 | | 0.002 | 0.003 | | 0.003 | 0.003 | | | |  |
| **MAR (weak)** |  |  |  |  | |  |  | |  |  | | | |  |
| Absolute Bias^a^ | 0.010 | 0.002 | 0.015 | 0.012 | | 0.029 | 0.011 | | 0.005 | 0.001 | | | |  |
| Relative Bias (%) | 10.235 | 2.172 | 14.571 | 11.624 | | 29.141 | 11.153 | | 5.331 | 1.051 | | | |  |
| Empirical SE | 0.090 | 0.064 | 0.056 | 0.057 | | 0.046 | 0.051 | | 0.051 | 0.054 | | | |  |
| Model-based SE | 0.090 | 0.063 | 0.060 | 0.060 | | 0.060 | 0.060 | | 0.060 | 0.060 | | | |  |
| Coverage (%) | 94.6 | 94.1 | 95.5 | 95.7 | | 96.7 | 98.0 | | 98.2 | 97.7 | | | |  |
| MSE | 0.008 | 0.004 | 0.003 | 0.003 | | 0.003 | 0.003 | | 0.003 | 0.003 | | | |  |
| **MAR (strong)** |  |  |  |  | |  |  | |  |  | | | |  |
| Absolute Bias^a^ | 0.023 | 0.008 | 0.023 | 0.018 | | 0.041 | 0.015 | | 0.006 | 0.002 | | | |  |
| Relative Bias (%) | 22.788 | 8.113 | 22.554 | 18.155 | | 41.413 | 15.231 | | 5.913 | 1.612 | | | |  |
| Empirical SE | 0.091 | 0.062 | 0.054 | 0.056 | | 0.043 | 0.048 | | 0.049 | 0.053 | | | |  |
| Model-based SE | 0.088 | 0.063 | 0.061 | 0.061 | | 0.060 | 0.061 | | 0.061 | 0.061 | | | |  |
| Coverage (%) | 94.3 | 95.0 | 95.9 | 96.2 | | 96.6 | 98.1 | | 98.1 | 97.6 | | | |  |
| MSE | 0.009 | 0.004 | 0.003 | 0.003 | | 0.004 | 0.003 | | 0.002 | 0.003 | | | |  |

Abbreviations: ACA, available case analysis; CCA, complete case analysis; continuous-calibration, imputation as a continuous variable using multivariate normal imputation with calibration; Empirical SE, empirical standard error; indicator-PDBR, indicator based imputation using multivariate normal imputation with projected distance-based rounding; MAR, missing at random; MCAR, missing completely at random; Model-based SE, model based standard error; MVNI, multivariate normal imputation; PMM, predictive mean matching; MSE, mean square error.

^a^Monte Carlo standard error did not exceed 0.003.

^b^True value for the mean difference in body mass index for age z-scores between ex-smokers and never-smokers is 0.10.

^c^ Methods in which the imputation models failed to converge are not shown.

^d^Minimal differences were observed between the results of predictive mean matching with 5 and 10 nearest observations. Therefore, only the results for this method with 5 nearest observations are presented.

^e^0.2%, 0.3%, and 0.3% of the datasets failed to converge when using indicator-PDBR without restrictions under MCAR, MAR (weak), and MAR (strong) scenarios respectively.

^f^0.1% of the datasets failed to converge when using indicator-PDBR with restrictions under the MAR (strong) scenario.

**Stata code for FCS with PMM imputation with restrictions**

cap log close

version 13.1

clear

set more off

log using MAR_pmm_S2_R.log, replace

clear

cd "/home_data/scratch/anurika/Simulation Study 2/48"

set seed 03012017

tempname MAR_pmm_S2_R

postfile `MAR_pmm_S2_R' Dataset Parameter_ex SE_ex LCL_ex UCL_ex Within_ex Between_ex Parameter_current SE_current LCL_current UCL_current Within_current Between_current using results_48, replace

forvalues k=1(1)1000 {

clear

use dataMAR_S2_`k'

**Before imputation modification

replace m_smoking_MAR1=0 if m_smoking_MAR5==0

replace m_smoking_MAR2=0 if m_smoking_MAR5==0

replace m_smoking_MAR3=0 if m_smoking_MAR5==0

replace m_smoking_MAR4=0 if m_smoking_MAR5==0

replace m_smoking_MAR1=0 if m_smoking_MAR4==0

replace m_smoking_MAR2=0 if m_smoking_MAR4==0

replace m_smoking_MAR3=0 if m_smoking_MAR4==0

replace m_smoking_MAR1=0 if m_smoking_MAR3==0

replace m_smoking_MAR2=0 if m_smoking_MAR3==0

replace m_smoking_MAR1=0 if m_smoking_MAR2==0

**Create temporary variable for binary logistic imputation

gen S10=m_smoking_MAR0

gen S11=m_smoking_MAR1

gen S12=m_smoking_MAR2

gen S13=m_smoking_MAR3

gen S14=m_smoking_MAR4

gen S15=m_smoking_MAR5

reshape long m_smoking m_depression bmiz prev_depression prev_smoking m_smoking_MAR scage S1, i(id) j(wave)

replace S1=. if S1==0 & wave!=0

recode S1 (1=0) (2=1) if wave!=0

reshape wide m_smoking m_depression bmiz prev_depression prev_smoking m_smoking_MAR scage S1, i(id) j(wave)

gen Sce10=1 if m_smoking0==1 | m_smoking0==2

gen Sce11=1 if m_smoking_MAR1==1 | m_smoking_MAR1==2

replace Sce11=. if Sce10==1

gen Sce12=1 if m_smoking_MAR2==1 | m_smoking_MAR2==2

replace Sce12=. if Sce10==1 | Sce11==1

gen Sce13=1 if m_smoking_MAR3==1 | m_smoking_MAR3==2

replace Sce13=. if Sce10==1 | Sce11==1 | Sce12==1

gen Sce14=1 if m_smoking_MAR4==1 | m_smoking_MAR4==2

replace Sce14=. if Sce10==1 | Sce11==1 | Sce12==1 | Sce13==1

*FCS imuptation Scenario 2

mi set flong

mi register imputed S10 S11 S12 S13 S14 S15

mi register regular scage* breastfeeding m_age m_education birthweight sex ses m_depression* bmiz*

mi impute chained (pmm, knn(10)) S11 S12 S13 S14 S15= S10 bmiz2 bmiz3 bmiz4 bmiz5 bmiz6 breastfeeding m_age m_education birthweight sex ses m_depression0 m_depression1 m_depression2 m_depression3 m_depression4 m_depression5 m_depression6, add(65)

mi reshape long m_smoking m_depression bmiz prev_depression prev_smoking m_smoking_MAR scage S1 Sce1, i(id) j(wave)

recode S1 (0=1) (1=2) if wave!=0

mi reshape wide m_smoking m_depression bmiz prev_depression prev_smoking m_smoking_MAR scage S1 Sce1, i(id) j(wave)

**Replace the m_smoking_MAR in the imputations with the imputed values according to the scenarios

replace m_smoking_MAR1=S11 if Sce10==1 & _mi_m!=0

replace m_smoking_MAR2=S12 if Sce10==1 & _mi_m!=0

replace m_smoking_MAR3=S13 if Sce10==1 & _mi_m!=0

replace m_smoking_MAR4=S14 if Sce10==1 & _mi_m!=0

replace m_smoking_MAR5=S15 if Sce10==1 & _mi_m!=0

replace m_smoking_MAR2=S12 if Sce11==1 & _mi_m!=0

replace m_smoking_MAR3=S13 if Sce11==1 & _mi_m!=0

replace m_smoking_MAR4=S14 if Sce11==1 & _mi_m!=0

replace m_smoking_MAR5=S15 if Sce11==1 & _mi_m!=0

replace m_smoking_MAR3=S13 if Sce12==1 & _mi_m!=0

replace m_smoking_MAR4=S14 if Sce12==1 & _mi_m!=0

replace m_smoking_MAR5=S15 if Sce12==1 & _mi_m!=0

replace m_smoking_MAR4=S14 if Sce13==1 & _mi_m!=0

replace m_smoking_MAR5=S15 if Sce13==1 & _mi_m!=0

replace m_smoking_MAR5=S15 if Sce14==1 & _mi_m!=0

**Create temporary variable for binary logistic imputation

gen S20=m_smoking_MAR0

gen S21=m_smoking_MAR1

gen S22=m_smoking_MAR2

gen S23=m_smoking_MAR3

gen S24=m_smoking_MAR4

gen S25=m_smoking_MAR5

*FCS imuptation Scenario 3

mi set flong

mi register imputed S20 S21 S22 S23 S24 S25

mi register regular scage* breastfeeding m_age m_education birthweight sex ses m_depression* bmiz*

mi impute chained (pmm, knn(10)) S21 S22 S23 S24 S25= S20 bmiz2 bmiz3 bmiz4 bmiz5 bmiz6 breastfeeding m_age m_education birthweight sex ses m_depression0 m_depression1 m_depression2 m_depression3 m_depression4 m_depression5 m_depression6, replace

**Replace the m_smoking_MAR in the imputations with the imputed values according to the scenarios

replace m_smoking_MAR1=S21 if m_smoking_MAR0==0 & m_smoking_MAR5==. & _mi_m!=0 & m_smoking_MAR1==.

replace m_smoking_MAR2=S22 if m_smoking_MAR0==0 & m_smoking_MAR5==. & _mi_m!=0 & m_smoking_MAR2==.

replace m_smoking_MAR3=S23 if m_smoking_MAR0==0 & m_smoking_MAR5==. & _mi_m!=0 & m_smoking_MAR3==.

replace m_smoking_MAR4=S24 if m_smoking_MAR0==0 & m_smoking_MAR5==. & _mi_m!=0 & m_smoking_MAR4==.

replace m_smoking_MAR5=S25 if m_smoking_MAR0==0 & m_smoking_MAR5==. & _mi_m!=0 & m_smoking_MAR5==.

replace m_smoking_MAR1=S21 if m_smoking_MAR0==0 & m_smoking_MAR5==1 & _mi_m!=0 & m_smoking_MAR1==. | m_smoking_MAR0==0 & m_smoking_MAR5==2 & _mi_m!=0 & m_smoking_MAR1==.

replace m_smoking_MAR2=S22 if m_smoking_MAR0==0 & m_smoking_MAR5==1 & _mi_m!=0 & m_smoking_MAR2==. | m_smoking_MAR0==0 & m_smoking_MAR5==2 & _mi_m!=0 & m_smoking_MAR2==.

replace m_smoking_MAR3=S23 if m_smoking_MAR0==0 & m_smoking_MAR5==1 & _mi_m!=0 & m_smoking_MAR3==. | m_smoking_MAR0==0 & m_smoking_MAR5==2 & _mi_m!=0 & m_smoking_MAR3==.

replace m_smoking_MAR4=S24 if m_smoking_MAR0==0 & m_smoking_MAR5==1 & _mi_m!=0 & m_smoking_MAR4==. | m_smoking_MAR0==0 & m_smoking_MAR5==2 & _mi_m!=0 & m_smoking_MAR4==.

replace m_smoking_MAR1=S21 if m_smoking_MAR0==0 & m_smoking_MAR4==1 & _mi_m!=0 & m_smoking_MAR1==. | m_smoking_MAR0==0 & m_smoking_MAR4==2 & _mi_m!=0 & m_smoking_MAR1==.

replace m_smoking_MAR2=S22 if m_smoking_MAR0==0 & m_smoking_MAR4==1 & _mi_m!=0 & m_smoking_MAR2==. | m_smoking_MAR0==0 & m_smoking_MAR4==2 & _mi_m!=0 & m_smoking_MAR2==.

replace m_smoking_MAR3=S23 if m_smoking_MAR0==0 & m_smoking_MAR4==1 & _mi_m!=0 & m_smoking_MAR3==. | m_smoking_MAR0==0 & m_smoking_MAR4==2 & _mi_m!=0 & m_smoking_MAR3==.

replace m_smoking_MAR1=S21 if m_smoking_MAR0==0 & m_smoking_MAR3==1 & _mi_m!=0 & m_smoking_MAR1==. | m_smoking_MAR0==0 & m_smoking_MAR3==2 & _mi_m!=0 & m_smoking_MAR1==.

replace m_smoking_MAR2=S22 if m_smoking_MAR0==0 & m_smoking_MAR3==1 & _mi_m!=0 & m_smoking_MAR2==. | m_smoking_MAR0==0 & m_smoking_MAR3==2 & _mi_m!=0 & m_smoking_MAR2==.

replace m_smoking_MAR1=S21 if m_smoking_MAR0==0 & m_smoking_MAR2==1 & _mi_m!=0 & m_smoking_MAR1==. | m_smoking_MAR0==0 & m_smoking_MAR2==2 & _mi_m!=0 & m_smoking_MAR1==.

replace m_smoking_MAR2=S22 if m_smoking_MAR1==0 & m_smoking_MAR5==1 & _mi_m!=0 & m_smoking_MAR2==. | m_smoking_MAR1==0 & m_smoking_MAR5==2 & _mi_m!=0 & m_smoking_MAR2==.

replace m_smoking_MAR3=S23 if m_smoking_MAR1==0 & m_smoking_MAR5==1 & _mi_m!=0 & m_smoking_MAR3==. | m_smoking_MAR1==0 & m_smoking_MAR5==2 & _mi_m!=0 & m_smoking_MAR3==.

replace m_smoking_MAR4=S24 if m_smoking_MAR1==0 & m_smoking_MAR5==1 & _mi_m!=0 & m_smoking_MAR4==. | m_smoking_MAR1==0 & m_smoking_MAR5==2 & _mi_m!=0 & m_smoking_MAR4==.

replace m_smoking_MAR2=S22 if m_smoking_MAR1==0 & m_smoking_MAR4==1 & _mi_m!=0 & m_smoking_MAR2==. | m_smoking_MAR1==0 & m_smoking_MAR4==2 & _mi_m!=0 & m_smoking_MAR2==.

replace m_smoking_MAR3=S23 if m_smoking_MAR1==0 & m_smoking_MAR4==1 & _mi_m!=0 & m_smoking_MAR3==. | m_smoking_MAR1==0 & m_smoking_MAR4==2 & _mi_m!=0 & m_smoking_MAR3==.

replace m_smoking_MAR2=S22 if m_smoking_MAR1==0 & m_smoking_MAR3==1 & _mi_m!=0 & m_smoking_MAR2==. | m_smoking_MAR1==0 & m_smoking_MAR3==2 & _mi_m!=0 & m_smoking_MAR2==.

replace m_smoking_MAR3=S23 if m_smoking_MAR2==0 & m_smoking_MAR5==1 & _mi_m!=0 & m_smoking_MAR3==. | m_smoking_MAR2==0 & m_smoking_MAR5==2 & _mi_m!=0 & m_smoking_MAR3==.

replace m_smoking_MAR4=S24 if m_smoking_MAR2==0 & m_smoking_MAR5==1 & _mi_m!=0 & m_smoking_MAR4==. | m_smoking_MAR2==0 & m_smoking_MAR5==2 & _mi_m!=0 & m_smoking_MAR4==.

replace m_smoking_MAR3=S23 if m_smoking_MAR2==0 & m_smoking_MAR4==1 & _mi_m!=0 & m_smoking_MAR3==. | m_smoking_MAR2==0 & m_smoking_MAR4==2 & _mi_m!=0 & m_smoking_MAR3==.

replace m_smoking_MAR4=S24 if m_smoking_MAR3==0 & m_smoking_MAR5==1 & _mi_m!=0 & m_smoking_MAR4==. | m_smoking_MAR3==0 & m_smoking_MAR5==2 & _mi_m!=0 & m_smoking_MAR4==.

mi reshape long m_smoking m_depression bmiz prev_depression prev_smoking m_smoking_MAR scage S1 S2 Sce1, i(id) j(wave)

sort _mi_m id wave

bysort _mi_m id: gen prev_smoking_MAR= m_smoking_MAR[_n-1]

mi estimate: xtmixed bmiz i.prev_smoking_MAR scage i.breastfeeding m_age i.m_education birthweight i.sex ses || id:

mi estimate, vartable

matrix coefficients=r(table)

scalar parameter_ex=coefficients[1,2]

scalar parameter_current=coefficients[1,3]

scalar standard_error_ex=coefficients[2,2]

scalar standard_error_current=coefficients[2,3]

scalar lcl_ex=coefficients[5,2]

scalar ucl_ex=coefficients[6,2]

scalar lcl_current=coefficients[5,3]

scalar ucl_current=coefficients[6,3]

matrix within=e(W_mi)

scalar within_ex=within[2,2]

scalar within_current=within[3,3]

matrix between=e(B_mi)

scalar between_ex=between[2,2]

scalar between_current=between[3,3]

post `MAR_pmm_S2_R' (`k') (parameter_ex) (standard_error_ex) (lcl_ex) (ucl_ex) (within_ex) (between_ex) (parameter_current) (standard_error_current) (lcl_current) (ucl_current) (within_current) (between_current)

}

postclose `MAR_pmm_S2_R'

log close

exit

**References**

1. Thoemmes F, Mohan K: **Graphical Representation of Missing Data Problems**. *Structural Equation Modeling: A Multidisciplinary Journal* 2015, **22**(4):631-642.

2. Carlin JB: **Multiple imputation: a perspective and historical overview**. In: *Handbook of Missing Data.* edn. Edited by Molenberghs G, Fitzmaurice GM, Kenward MG, Tsiatis AA, Verbeke G. Boca Raton: Chapman & Hall/CRC Press; 2015.

3. Allison PD: **Missing data:** Thousand Oaks, Calif.: SAGE Publications, London; 2002.

4. Lee KJ, Galati JC, Simpson JA, Carlin JB: **Comparison of methods for imputing ordinal data using multivariate normal imputation: a case study of non-linear effects in a large cohort study**. *Statistics in Medicine* 2012, **31**(30):4164-4174.

5. Galati JC, Seaton KA, Lee KJ, Simpson JA, Carlin JB: **Rounding non-binary categorical variables following multivariate normal imputation: evaluation of simple methods and implications for practice**. *Journal of Statistical Computation and Simulation* 2012, **84**(4):798-811.

6. Yucel RM, He Y, Zaslavsky AM: **Using Calibration to Improve Rounding in Imputation**. *The American Statistician* 2008, **62**(2):125-129.

7. Yucel RM, He Y, Zaslavsky AM: **Gaussian-based routines to impute categorical variables in health samplings**. *Statistics in Medicine* 2011, **30**(29):3447-3460.

8. White IR, Royston P, Wood AM: **Multiple imputation using chained equations: Issues and guidance for practice**. *Statistics in Medicine* 2011, **30**(4):377-399.

9. Welch C, Bartlett J, Peterson I: **Application of multiple imputation using the two-fold fully conditional specification algorithm in longitudinal clinical data**. *The Stata Journal* 2014, **14**(2):418-431.

10. Welch C, Petersen I, Bartlett JW, White IR, Marston L, Morris RW, Nazareth I, Walters K, Carpenter J: **Evaluation of two-fold fully conditional specification multiple imputation for longitudinal electronic health record data**. *Statistics in Medicine* 2014, **33**(21):3725-3737.
